# Fibroblast Growth Factor Type 2 Signaling Is Critical for DNA Repair in Human Keratinocyte Stem Cells

**DOI:** 10.1002/stem.485

**Published:** 2010-09

**Authors:** Ghida Harfouche, Pierre Vaigot, Walid Rachidi, Odile Rigaud, Sandra Moratille, Mélanie Marie, Gilles Lemaitre, Nicolas O Fortunel, Michèle T Martin

**Affiliations:** a CEA, iRCM, Laboratory of Genomics and Radiobiology of Keratinopoiesis2 Rue G. Crémieux, 91057, Evry cedex, France; b INAC/SCIB/LAN, CEA de Grenoble17 Rue des Martyrs, 38054, Grenoble, France; c Cognis, Laboratoires Sérobiologiques3 rue de Seichamps, Pulnoy, France; d INSERM/UEVE U861, I-STEM, AFM5 rue H. Desbruères, Evry, France

**Keywords:** FGF2, Stem cells, Progenitors, Keratinocyte, Human skin, DNA repair

## Abstract

Tissue stem cells must be endowed with superior maintenance and repair systems to ensure genomic stability over multiple generations, which would be less necessary in more differentiated cells. We previously reported that human keratinocyte stem cells were more resistant to ionizing radiation toxicity than their direct progeny, the keratinocyte progenitor cells. In the present study we addressed the mechanisms underlying this difference. Investigations of DNA repair showed that both single and double DNA strand breaks were repaired more rapidly and more efficiently in stem cells than in progenitors. As cell signaling is a key regulatory step in the management of DNA damage, a gene profiling study was performed. Data revealed that several genes of the fibroblast growth factor type 2 (FGF2) signaling pathway were induced by DNA damage in stem cells and not in progenitors. Furthermore, an increased content of the FGF2 protein was found in irradiated stem cells, both for the secreted and the cellular forms of the protein. To examine the role of endogenous FGF2 in DNA repair, stem cells were exposed to FGF2 pathway inhibitors. Blocking the FGF2 receptor (FGF receptor 1) or the kinase (Ras-mitogen-activated protein kinase 1) resulted in a inhibition of single and double DNA strand-break repair in the keratinocyte stem cells. Moreover, supplementing the progenitor cells with exogenous FGF2 activated their DNA repair. We propose that, apart from its well-known role as a strong mitogen and prosurvival factor, FGF2 helps to maintain genomic integrity in stem cells by activating stress-induced DNA repair. Stem Cells 2010; 28:1639–1648.

## INTRODUCTION

Fibroblast growth factors (FGFs) are one of the largest growth factor families, comprising 22 members with 13%–71% sequence similarity in mammals [1]. FGF type 2 (FGF2), or basic FGF, is a prototype member of the family. It interacts with high-affinity receptors (FGF receptors [FGFRs]), which are transmembrane tyrosine kinases; the FGFR1c isoform being its prime target. The binding of FGF2 to FGFR1 induces receptor autophosphorylation on several tyrosine residues, which in turn activates downstream effector molecules, leading to the activation of the Ras-mitogen-activated protein kinase (MAPK) cascade [2]. This cascade promotes translocation of MAPKs to the nucleus, where they phosphorylate and directly activate specific target proteins, including transcription factors.

FGF2 signaling is critical in governing stem cell functions. It is a major regulator of self-renewal for both human embryonic stem cells [3–5] and induced pluripotent stem cells [6,7]. Of all the growth factors tested in tissue culture, FGF2 most strongly promotes human embryonic stem cell self-renewal, which requires activation of transforming growth factor/Activin/Nodal pathways and repression of bone morphogenetic protein/growth differentiation factor pathways. FGF2 is also highly expressed in various somatic cell types where it has an intrinsic function in the regulation of cell proliferation, differentiation, and survival. It also regulates self-renewal and immaturity of many tissue-specific stem cells, including cells from the mouse striatum [8–10], bone marrow mesenchymal stem cells [11], and adipose tissue-derived stem cells [12,13].

FGF2 also has a protective function against stress-induced cell death, which has been described in various types of stem cells. In human embryonic stem cells, FGF2 protects against oxidative and radiation stress [5]. Intestinal crypt survival is increased by FGF2 after radiation damage, which indirectly shows protection of intestinal stem cells [14,15]. In the rat cortex, it was proposed that FGF2 protects neurogenesis, based on the observations that FGF2 is released in response to apoptotic insults such as hypoxia [16], and that the conditioned medium from the apoptotic cortex stimulates the number of BrdU-incorporating nuclei in cultured stem cells isolated from the subventricular zone [17]. In mesenchymal stem cells, improved survival under hypoxia was found after transfection with the FGF2 gene [18]. In contrast to this well-described role of protection against cell death, very few studies have investigated the relationships between FGF2, DNA repair and genomic stability in stem cells, and notably in epithelial stem cells.

Keratinocyte stem cells are undifferentiated and quiescent cells, responsible for the long-term maintenance of the epidermis, a tissue in perpetual renewal [19,20]. The direct stem cell progeny, called keratinocyte progenitor or transient amplifying cells, are also located in the basal layer of the epidermis, where they divide to produce more differentiated cells that migrate to the upper layers. Keratinocyte progenitors are thus responsible for short-term maintenance of the epidermis. Although no specific marker for keratinocyte stem cells has been found, a combination of markers was identified that allows enrichment of these cells from a population of keratinocytes. Specifically, an adhesion molecule (CD49f, α6 integrin, Itg-α6) combined with a proliferation-associated marker (CD71, transferrin receptor, Trf-R) was associated with enrichment of cell populations in stem and progenitor cells from the same skin sample by flow cytometry [21,22]. Using this phenotype, we recently demonstrated that a cell population enriched in human keratinocyte stem cells (Itg-α6^bri^/Trf-R^dim^) was more resistant to γ-rays than a corresponding population enriched in keratinocyte progenitors (Itg-α6^bri^/Trf-R^bri^) [23]. Our previous results also showed that the most significant up regulated network in irradiated stem cells comprised cytokines and growth factors, including FGF2. The present study investigated the role of FGF2 in the stress response of primary cultures of keratinocyte stem and progenitor cells. The stem cells demonstrated activated DNA repair activity, and the induction of endogenous FGF2 signaling was shown to be critical for the repair of DNA damage. Thus, keratinocyte stem cells use enhanced DNA damage signaling and repair to protect their genome.

## MATERIALS AND METHODS

### Reagents

Dulbecco's modified Eagle's medium (DMEM) was from Invitrogen (Cergy-Pontoise, France, http://www.invitrogen.com), semidefined KGM2 medium from Clonetics-Lonza (Basel, Switzerland, http://www.lonza.com) and the fetal calf serum from Hyclone-Thermo Fischer Scientific (Waltham, MA, USA, http://www.hyclone.com). The antibiotics (10,000 U/ml penicillin G sodium and 10,000 μg/ml streptomycin sulfate in 0.85% saline) were from Gibco-Invitrogen (Cergy-Pontoise, France, http://www.Invitrogen.com). All other reagents were from Sigma-Aldrich (St Louis, MO, USA, http://www.sigmaaldrich.com).

### Isolation of Keratinocytes

This study was approved by the review board of the iRCM (Institut de Radiobiologie Cellulaire et Moléculaire, CEA, Fontenay-aux-Roses, France), and is in accordance with the scientific, ethical, safety, and publication policy of CEA (CODECO number DC-2008-228, reviewed by the ethical research committee IDF-3). Neonatal foreskin samples were obtained after informed consent of the patient's parents. Skin was incubated overnight at 4°C in a solution containing DMEM, dispase 2.4 U/ml (Invitrogen) and trypsin 0.5 g/l (Gibco). Subsequently, the dermis and epidermis were separated using a pair of forceps, and the epidermis was incubated in trypsin 0.05% EDTA for 20 minutes at 37°C.

### Cell staining, Sorting, and Irradiation

Keratinocyte populations were isolated from fresh preparations using two cell-surface markers, α6-integrin (Itg-α6) and transferrin receptor (Trf-R). Streptavidin allophycocyanin mouse anti-human CD71 Mab (Trf-R), and R-phycoerythrin-conjugated rat anti-human CD49f Mab (Itg-α6) (Pharmigen Becton Dickinson, Franklin Lakes, NJ, USA, http://www.bd.com) were used according to a published protocol [21] and as described previously [23]. This staining method enabled the separation of one population enriched in stem cells (Itg-α6^bri^/Trf-R^dim^) and another enriched in progenitors (Itg-α6^bri^/Trf-R^bri^) by flow cytometry. Equivalent numbers of sorted cells from each population were plated onto Biocoat cellware rat-tail collagen type-I culture dishes in a serum-free growth medium (KGM2) and incubated overnight at 37°C. Seventeen hours after plating, the cells were exposed to a dose of 2 Gy using ^137^Cs sources (γ-rays, IBL637) at a rate of 0.6 Gy/minute.

### Alkaline Comet Assay

The DNA damage was assessed using the alkaline comet assay according to the procedure previously described [24]. This assay allows the detection of single and double-strand breaks as well as alkali-labile sites expressed as frank single-strand breaks in individual cells [25]. The slides were stained with ethidium bromide (10 μg/ml) and comet analysis was performed using the image analysis Komet six software (Kinetic Imaging Ltd, Andor Technology plc. Belfast, Ireland, http://www.andor-tech.com). The tail moment, defined as the product of the tail length by the ratio of the fluorescence intensity between the head and the tail, was taken as the index of damage. For each treatment, the average tail moment was determined from the analysis of 450 comets (triplicate slides, 150 comets analyzed per slides).

### Histone H2AX Phosphorylated on Serine 139 Assay

Cells from each sorted population were plated onto Biocoat type-I four-well culture slides (Becton Dickinson) in KGM2 medium. The next day, the cells were exposed to a 2 Gy radiation dose and fixed with 100% ice-cold methanol 5-minute, 15-minute, 30-minute, 4-hour, and 24-hour postirradiation. The fixed cells were dipped in PBS with 10% goat serum. The slides were then incubated with human monoclonal anti-phospho-histone H2AX (Ser139) (Upstate, Millipore, Molsheim, France, http://www.upstate.com) for 1 hour at room temperature before being exposed to Cy3-conjugated goat anti-mouse secondary antibody for 45 minutes in the dark. Finally, the slides were mounted using Vectashield hard-set mounting medium with 4–6 diamidino-2-phenylindol-2-HCl (Vector Laboratories, Burlingame, CA, USA, http://www.vectorlabs.com). Fluorescence images were captured on a Zeiss Axioplan two imager (Zeiss, Oberkochen, Germany, http://www.zeiss.com) with a Plan Apochromat oil-immersion lens (×63 magnification; numerical aperture, 1.4). An AxioCam HRC camera and AxioVision software, version 4.6, were used to capture the images. For quantitative analysis, foci were manually counted under the microscope and imaging was performed using a ×63 lens. For each time point, approximately 100 cells per population were counted.

### Cell Cycle Analysis

Cell cycle profiles were analyzed in two sorting gates corresponding to keratinocyte stem cells and progenitors, as previously described [23]. For analyses at the time of cell sorting, the total keratinocyte population isolated from the epidermis was incubated with Hoechst 33,342 (20 μg/ml) for 1 hour at 37°C under gentle shaking and was then immediately analyzed by flow cytometry. For cultured cells, 10^5^ sorted keratinocyte stem cells and progenitors were plated onto collagen type-I culture dishes in KGM2 growth medium and incubated overnight. The next day, the cells were either exposed to 2 Gy or were sham-irradiated. Twenty-four hours after exposure, the cells were collected by trypsinization, resuspended in PBS, and fixed in 70% ethanol. The cells were washed with PBS, treated with 40 μg/mL RNase for 20 minutes at 37°C, and then propidium iodide (20 μg/ml) was added. To characterize the initiation of cell proliferation, keratinocytes were incubated with 10 μM bromodeoxyuridine (BrdU) for 20 minutes at 37°C. After washing with PBS, the cells were trypsinized at 72 hours after irradiation and processed using the BrdU Flow Kit (Pharmingen Becton Dickinson, San Diego, CA, USA, http://www.bdbiosciences.com/index_us.shtml) according to the manufacturer's instructions.

### Gene Profiling

Gene expression profiling was performed using the DNA microarray technology as described by Rachidi et al. [23]. These 50–53 bp oligonucleotides arrays were spotted with 26,068 human probes representing 21,000 genes (available in the MEDIANTE database (http://www.microarray.fr:8080/mediante). The expression level change was measured in stem and progenitor cells 3 hours after irradiation. In the modulated genes, the most significant biological pathways were identified by Ingenuity Pathway Analysis software (http://www.ingenuity.com). A complete description of the microarrays used in this study is available in the GEO database (http://www.ncbi.nlm.nih.gov/geo/) under the GEO accession no. GSE7693.

### Quantitative Reverse Transcription Polymerase Chain Reaction Analysis

Three hours after radiation exposure, total RNA was extracted from the two populations using the PicoPure Isolation kit (Arcturus, Alphelys, Plaisir, France, http://www.alphelys.com), following the manufacturer's protocol, with the optional DNase treatment step. The RNA quality was assessed in an Agilent 2100 bioanalyzer and an RNA 6000 Nano labChip kit (Agilent Technologies, Palo Alto, CA, USA, http://www.agilent.com; Supporting Information Fig. 1). Total extracted RNA was directly used for the reverse transcription polymerase chain reaction (RT-PCR) analysis without proceeding to any RNA amplification. This allowed the 18S rRNA to be used as an endogenous control in the RT-PCR analysis. Equivalent quantities of total RNA from each population were reverse-transcripted to cDNA (Superscript II Reverse Transcriptase, Invitrogen) in the presence of random primers (100 ng/μl), dNTP mix (10 mM each), 5× first strand buffer, DTT (0.1 M), and Superscript II enzyme. Each cDNA template (5–10 ng) was used in the PCR reactions with gene-specific primers (listed in the Supporting Information Table 1). Real-time PCR was performed in an ABI PRISM 7,500 Fast real-time PCR system (Applied Biosystems, Villebon-sur-Yvette, France, http://www.appliedbiosystems.com) using SYBR Green PCR Master Mix (Applied Biosystems) according to the protocol supplied by the kit's manufacturer.

### FGF2 Enzyme-Linked Solid-Phase Immunosorbent Assay

To quantify secreted and intracellular FGF2 protein, sorted keratinocytes were plated in KGM2 without the bovine pituitary extract fraction, and irradiated 18 hours later. At 72 hours after exposure, the supernatants were stored at −80°C and the cells were harvested in a RIPA protein lysis buffer to allow protein solubilization (Sigma-Aldrich). Cell protein contents were determined with a micro Protein Assay Kit (BCA, Thermo Scientific, Rockford, IL, USA, http://www.thermoscientific.com). The FGF2 contents were determined with an ELISA kit (R&D Systems Inc., Minneapolis, MN, USA, http://www.rndsystems.com) using 100 μl of conditioned medium (pure) or cell extracts (dilution 1/10), according to the manufacturer's instructions. The levels of FGF2 were measured at 450 nm with an ELISA plate reader (Spectramax 384, Molecular Devices, Sunnyvale, CA, USA, http://www.moleculardevices.com). For each experiment, the concentrations of the growth factor in the samples were determined from a standard curve for recombinant FGF2. The absolute FGF2 protein levels were then calculated with regard to the total protein contents found in the BCA assay.

### Blocking the FGF2 Pathway

The FGF2 pathway was blocked at the MAPK1 level using 5 μg/ml UO126 inhibitor (Upstate, Millipore), and at the receptor level (FGFR1) using 5 μg/ml mouse anti-FGFR1 monoclonal antibody (Chemicon International, Millipore). The inhibitor and the blocking antibody were added 30 minutes before radiation exposure. The specificity of the antibody effect was checked using unrelated IgM antibodies (Supporting Information Fig. 2).

### Supplementation of FGF2 on Progenitors

Human recombinant FGF-basic (FGF2, 10 ng/ml; Peprotech, Rocky Hill, NJ, USA, http://www.peprotech.com) was added to the progenitor cell growth medium 1 or 3 hours before radiation exposure. The histone H2AX phosphorylated on serine 139 (γH2AX) foci were then measured at 5 minutes, 15 minutes, 4 hours, and 24 hours postirradiation.

### Statistical Analysis

All of the statistical studies were performed using the Student's *t*-test. Differences were considered significant when the *p* value was < .05. The number of individuals in each experiment and the number of independent experiments are indicated in the respective figure legends.

## RESULTS

### Global DNA Damage Is Repaired More Rapidly in Keratinocyte Stem Cells Than in Progenitor Cells

**Figure 1:**
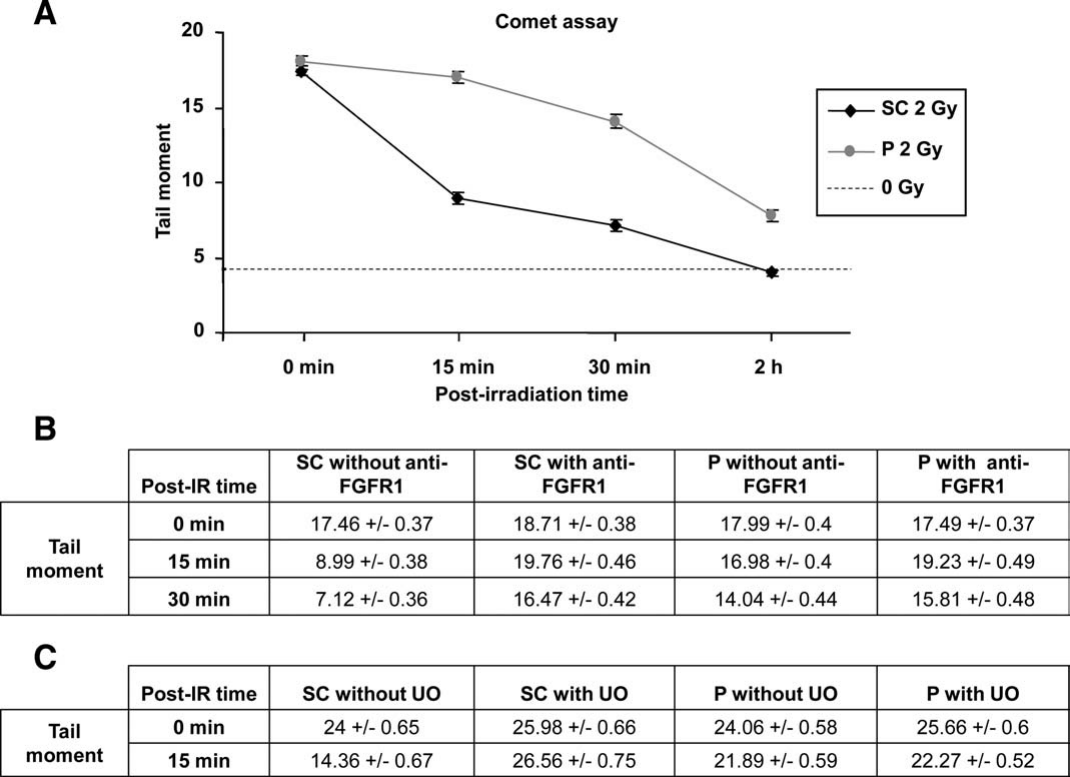
Global DNA damage is repaired more rapidly in keratinocyte stem cells. The alkaline comet assay was performed on irradiated keratinocytes to measure the repair of global DNA damage. Mean tail moments (arbitrary unit) are shown as a function of time after radiation exposure for three independent experiments. For each condition, three replicate slides were used, and 150 cells/slide were analyzed. *, *p* < .01. **(A)**: Repair kinetics of global DNA damage. Stem cells exhibited faster repair than progenitors and recovered to the baseline of nonirradiated cells at 2-hour postirradiation. The dashed line represents the mean tail moment for control stem and progenitor cells. **(B, C)**: The comet assay was performed after blocking the FGF2 pathway at the FGFR1 level using a blocking antibody **(B)**, and at the MAPK1 level using the UO126 inhibitor **(C)**. Both blockades inhibited DNA repair in stem cells. The assays shown in **(A–C)** were performed on keratinocytes from different donors. Abbreviations: FGFR1, fibroblast growth factor receptor 1; IR, ionizing radaition; P, progenitor cell; SC, stem cell; UO, UO126 inhibitor.

The global DNA damage and its repair were characterized in sorted populations enriched for stem cells and progenitors using the alkaline comet assay (Fig. 1A). This assay particularly evaluates the repair of DNA single-strand breaks, which are one of the most frequent lesions induced by ionizing radiation. The sham-irradiated cells from stem cells and progenitors presented tail moments between 0 and 5, with a mean of 3.37 for control stem cells and 4.84 for control progenitors. Directly after exposure to 2 Gy (0 minutes), the mean tail moments were similar in the two populations (17.46 for stem cells and 17.99 for progenitor cells), indicating that the induced damage was similar. However, 15 minutes postexposure, the irradiated stem cells presented a decreased tail moment (8.99), indicating damage repair, whereas the tail moment in the progenitor cells remained almost unchanged (16.98). Two hours postirradiation, the stem cells had recovered the mean tail moment of sham-irradiated cells (3.96), whereas the repair process was not yet completed in the progenitors (7.8). These results show that the keratinocyte stem cells exhibited a faster repair of global DNA damage, particularly of DNA single-strand breaks, than the progenitor cells.

### DNA Double-Strand Breaks Are Repaired More Rapidly in Keratinocyte Stem Cells Than in Progenitor Cells

**Figure 2:**
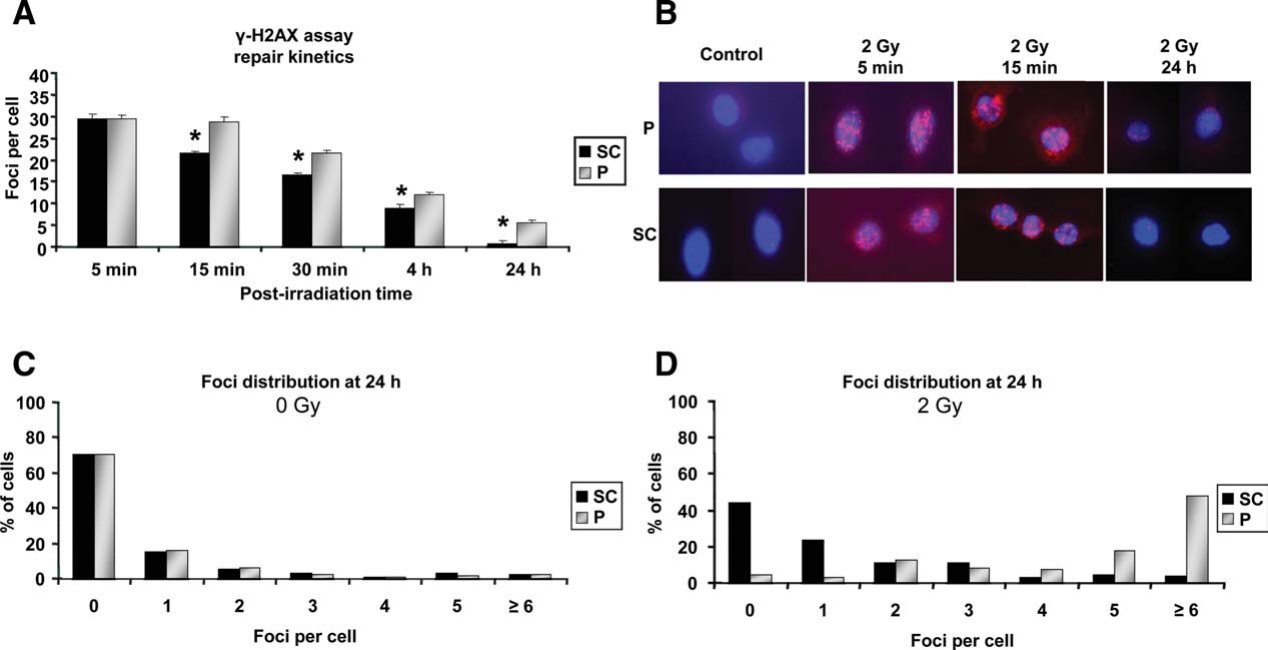
DNA double-strand breaks are repaired more rapidly and more efficiently in stem cells than in progenitor cells. **(A)**: Stem cells exhibited a more rapid DNA repair than progenitor cells. The γH2AX assay, based on the detection of histone H2AX phosphorylated on Ser 139, characterized the repair of DNA double-strand breaks in irradiated keratinocytes. Four to ten independent experiments on skin samples from different donors were performed for each time point and 100 cells per time point were counted for each experiment. *, *p* < .01. **(B)**: Fluorescence images of cells labeled with 4–6 diamidino-2-phenylindol-2-HCl (blue) for the nuclei and with Cy3 (red) for the γH2AX foci. **(C**, **D)**: Double-strand break repair was more efficient in stem cells than in progenitor cells. The distribution of the number of γH2AX foci per cell was characterized at 24 hours after exposure in the control **(C)** and irradiated cells **(D)**. Most control cells had less than four foci per nucleus (SC: 94%; P: 95%) (*n* = 3). After 2 Gy, 73% of the progenitors presented at least four foci per nucleus, as compared with 10% for stem cells (*n* = 4). In the progenitors, the mean number of γH2AX foci was seven foci per nucleus. The last column shows the percentage of cells with the highest number of foci (≥6), ranging from 6 to 11 foci per cell. Abbreviations: γH2AX, histone H2AX phosphorylated on serine 139; P, progenitor cell; SC, stem cell.

The DNA double-strand breaks are the most deleterious type of radiation-induced damage. Thus, the γH2AX assay was performed to characterize the repair of DNA double-strand breaks in irradiated keratinocytes. This assay is based on the detection of rapid phosphorylation on serine 139 in the histone H2AX proteins located in the chromatin surrounding a double-strand break. Five minutes after exposure, the keratinocyte stem cells and the progenitor cells displayed a similar number of foci per cell, indicating similar initial radiation damage. After 15 minutes, the number of foci per cell had significantly decreased only in the stem cells (Fig. 2A, 2B). A faster decrease of foci in the stem cells and a difference in foci number between the two populations was observed all along the kinetics, indicating that double-strand break repair was more rapid in the stem cells than in the progenitor cells. Twenty-four hours after exposure, the number of γH2AX foci in irradiated stem cells had returned to a level similar to that found in the controls (Fig. 2C, 2D), whereas the progenitors still exhibited a mean of seven foci per nucleus (Fig. 2D). These results indicated that double-strand break repair was more efficient in the stem cells than in the progenitor cells. The number of residual foci at 24 hours is reported to be related to cell radiosensitivity, as cells exhibiting more than three foci are primed to cell death [26]. In the present study, 73% of the progenitors presented more than four foci per nucleus at 24 hours after exposure, as compared with 10% of stem cells. This percentage of progenitor cells immediately primed to cell death was very close to that found previously by evaluating late radiation toxicity using the standard cell survival assay: 2 Gy induced 71% death after 2 weeks of culture for progenitor colonies, versus 18% for the stem cells [23]. Thus, both immediate and late assays revealed stem cell radioresistance, in contrast to the high sensitivity for progenitor cells.

### Sorted Keratinocytes Are in the G0/G1 Phase of the Cell Cycle at the Time of Irradiation

**Figure 3:**
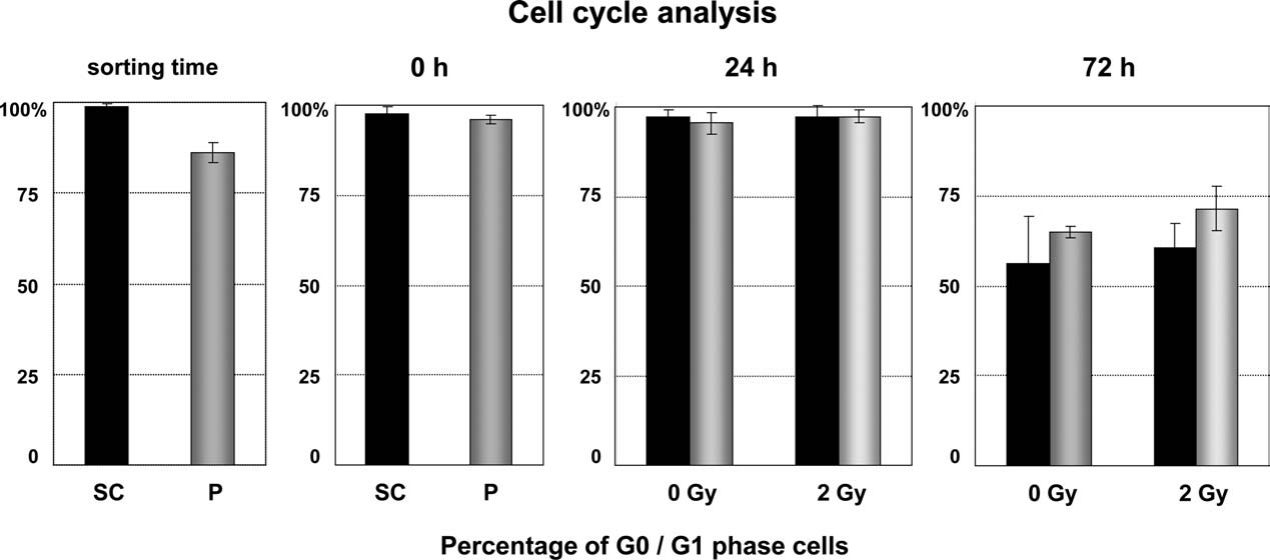
Cell cycle analysis of keratinocyte populations. Cell cycle analysis of the stem cells (SC, black bars) and progenitor cells (P, gray bars) was performed at four time points: at the time of cell sorting (time of sorting **[A]**); after overnight cell culture, at the time of irradiation (0 hour **[B]**); at 24 **(C)** and 72 hours **(D)** after irradiation with 2 Gy. The histograms show the mean percentage of G0/G1 phase cells ± SD, obtained from at least three independent experiments. Abbreviations: P, progenitor cell; SC, stem cell.

Mammalian cells use two major pathways to repair DNA double-strand breaks: nonhomologous end-joining (NHEJ), which is efficient in any phase of the cell cycle, and homologous recombination, which is only efficient in the S and G2 phases. Cell cycle analysis was thus performed using flow cytometry on the two sorting gates corresponding to keratinocyte stem cells and progenitors. At the time of cell sorting, 13.9% ± 2.8% of the progenitors isolated from the epidermis were in the S and G2/M phases of the cell cycle, against only 1.1% ± 0.9% of the stem cells (Fig. 3), thus confirming the higher proportion of actively cycling cells in progenitors as previously reported [23]. The next day after cell plating, at the time of irradiation (Fig. 3B), both populations were mainly in the G0/G1 phase of the cell cycle: 97.8% ± 1.9% of the stem cells, and 96.2% ± 1.2% of the progenitors. This result showed that the progenitors that adhered to the culture flask after plating consisted of the cells that were not actively cycling (86% of the total progenitor population), whereas the small fraction of actively cycling progenitors (13.9%) was lost. Twenty-four hours after radiation exposure, the cycle status remained unchanged for all conditions. Concerning DNA double-strand breaks, these data support the involvement of the NHEJ pathway in the repair processes characterized within the 24 hours time course of the present study.

At 72-hour postradiation exposure, the percentage of control cells in the G0/G1 phase was highly decreased for both stem cells (56.3% ± 12.8%) and progenitors (65% ± 1.7%), indicating that the control keratinocytes had re-entered the cell cycle. Interestingly, irradiated keratinocytes resumed proliferation similarly to the control cells (Fig. 3), which was confirmed by BrdU staining at 72 hours, where similar distributions in the cell cycle phases were found for all conditions (Supporting Information Fig. 3).

### FGF2 Expression Is Activated by DNA Damage in Keratinocyte Stem Cells

**Figure 4:**
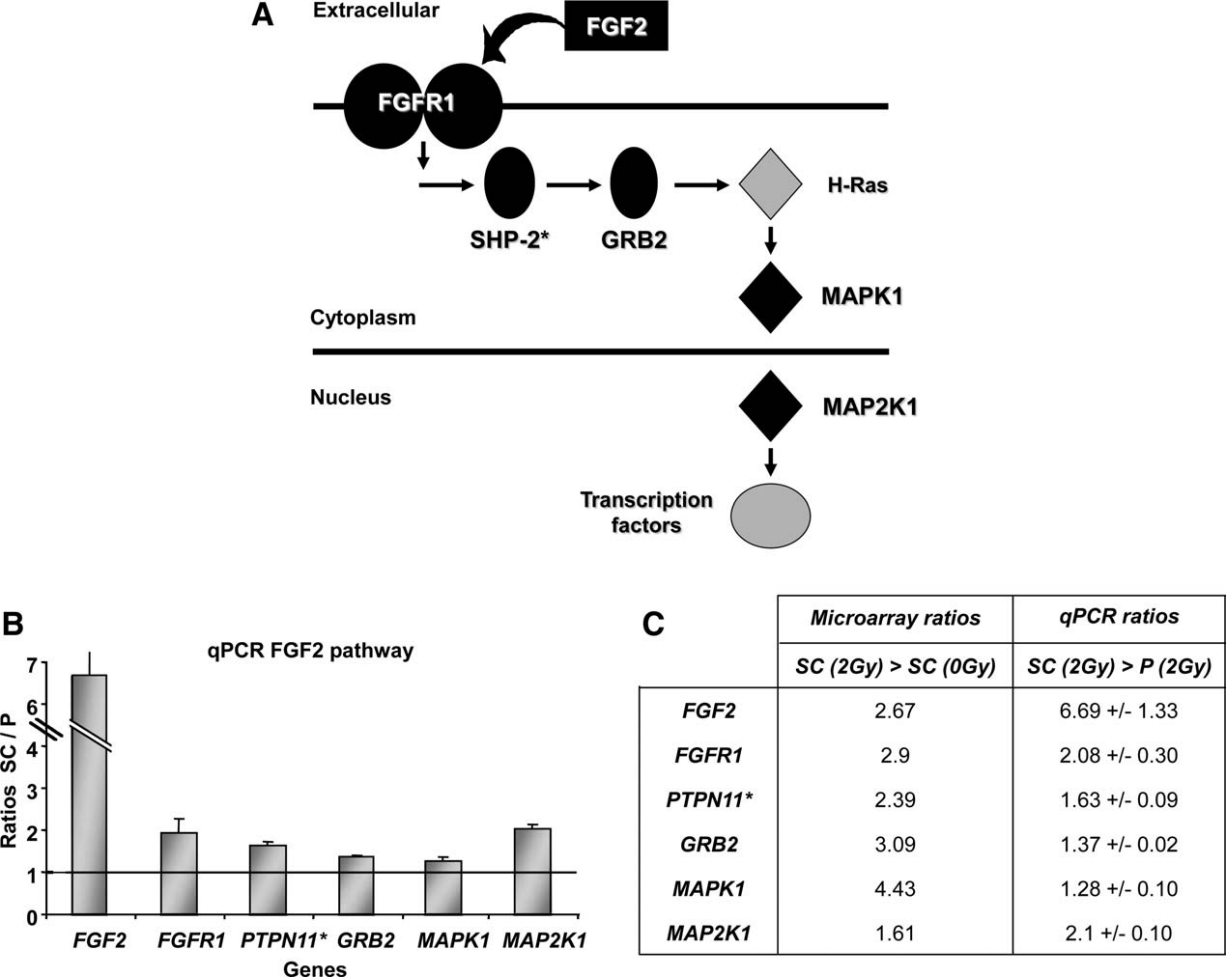
DNA damage induces the FGF2 signaling pathway in keratinocyte stem cells. **(A)**: Gene profiling showed that six genes of the FGF2 signaling pathway were found to be induced in irradiated versus control stem cells. The proteins corresponding to these genes appear in black in the FGF2 pathway. *SHP-2 is the PTPN11 gene product. **(B)**: Gene expression for the FGF2 pathway was compared in irradiated stem cells versus progenitor cells using qPCR. As the total RNA was not amplified, the 18S gene was used to normalize mRNA expression. The data shown were obtained from independent experiments performed on skin samples from four donors. **(C)**: Ratios of expression obtained with microarrays and qPCR between for six genes of the FGF2 pathway. *, *p* < .01. Abbreviations: FGF2, fibroblast growth factor type 2; FGFR1, fibroblast growth factor receptor 1; GRB2, growth factor receptor-bound protein 2; MAPK1, Ras-mitogen-activated protein kinase 1; MAPK2K1, Ras-mitogen-activated protein kinase 2K1; P, progenitor cell; qPCR, quantitative polymerase chain reaction; SC, stem cell; SHP-2, protein tyrosine phosphatase Shp2.

**Figure 5:**
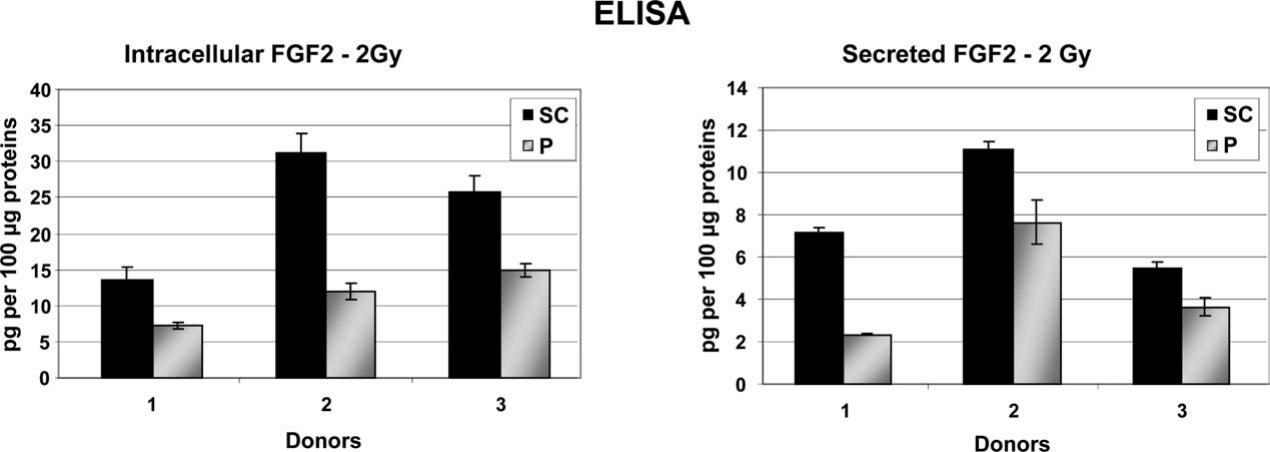
DNA damage increases FGF2 protein levels in irradiated stem cells. After radiation exposure, the FGF2 protein was increased in keratinocyte stem cells compared with progenitor cells, both in cells **(A)** and supernatants **(B)**. Seventy-two hours after 2 Gy, the cells and supernatants were harvested and the levels of FGF2 were determined using ELISA. The experiment was performed in triplicate on samples from four different skin donors. The histograms show the amount of FGF2 in pg (± SE) per 100 μg of the total proteins found in the corresponding cellular fraction with the BCA kit. The difference between the two cell populations was statistically significant both for secreted FGF2 in the supernatants (*p* = .034) and the cellular form of FGF2 in cell extracts (*p* = .019). Abbreviations: ELISA, enzyme-linked immunosorbent assay; FGF2, fibroblast growth factor type 2; P, progenitor cell; SC, stem cell.

To further characterize the response of keratinocyte stem cells to DNA damage, gene expression in irradiated and control cells was compared by global transcriptome analysis, which revealed a subset of 577 radiation-induced genes (1.5- to 13-fold change; Supporting Information Table 2). Ingenuity pathways analysis revealed that the most significantly upregulated network was related to cytokines and growth factors, including the FGF2. Figure 4A shows (in black) the components of the FGF2 pathway that were found to be upregulated in irradiated stem cells. Quantitative RT-PCR showed that irradiation induced at least a twofold increase of FGF2, FGFR1, and MAP2K1 mRNA levels in keratinocyte stem cells as compared with progenitors (Fig. 4B, 4C). Furthermore, the effects of radiation exposure were characterized at the FGF2 protein level (Fig. 5). Although FGF2 contents showed variations between the different skin donors, they were always increased in irradiated stem cells versus the progenitors, and the difference was statistically significant both for secreted FGF2 in the supernatants (*p* = .034) and the cellular form of FGF2 in cell extracts (*p* = .019). The mean FGF2 protein for all samples was increased by threefold in the cellular fraction and by 2.5-fold in the supernatants of stem cells compared with the progenitors. These data show that increased FGF2 mRNA and proteins are part of the stress response of the stem cell-enriched fraction of the human epidermis.

### FGF2 Regulates Keratinocyte Stem Cell Repair of Global DNA Damage

To determine whether or not FGF2 expression was related to the faster repair of global DNA damage observed in the stem cells, 5 μg/ml of a specific anti-FGFR1 antibody was added 30 minutes before radiation exposure to block FGF2 signaling. In the stem cells, the receptor level blockade impeded the decrease in the mean tail moment at 15 and 30 minutes after exposure, indicating an inhibition of the repair of global DNA damage in stem cells (Fig. 1B), whereas it had no impact on DNA repair in progenitor cells. When the FGF2 pathway was inhibited downstream of the receptor, using the specific MAPK1 inhibitor UO126 [27], DNA repair was also blocked in irradiated stem cells (Fig. 1C). These results showed that the FGF2 pathway blockade at the membrane or cytoplasm level inhibited global DNA repair in the stem cells, and notably the repair of DNA single-strand breaks.

### FGF2 Regulates Keratinocyte Stem Cell Repair of DNA Double-Strand Breaks

**Figure 6:**
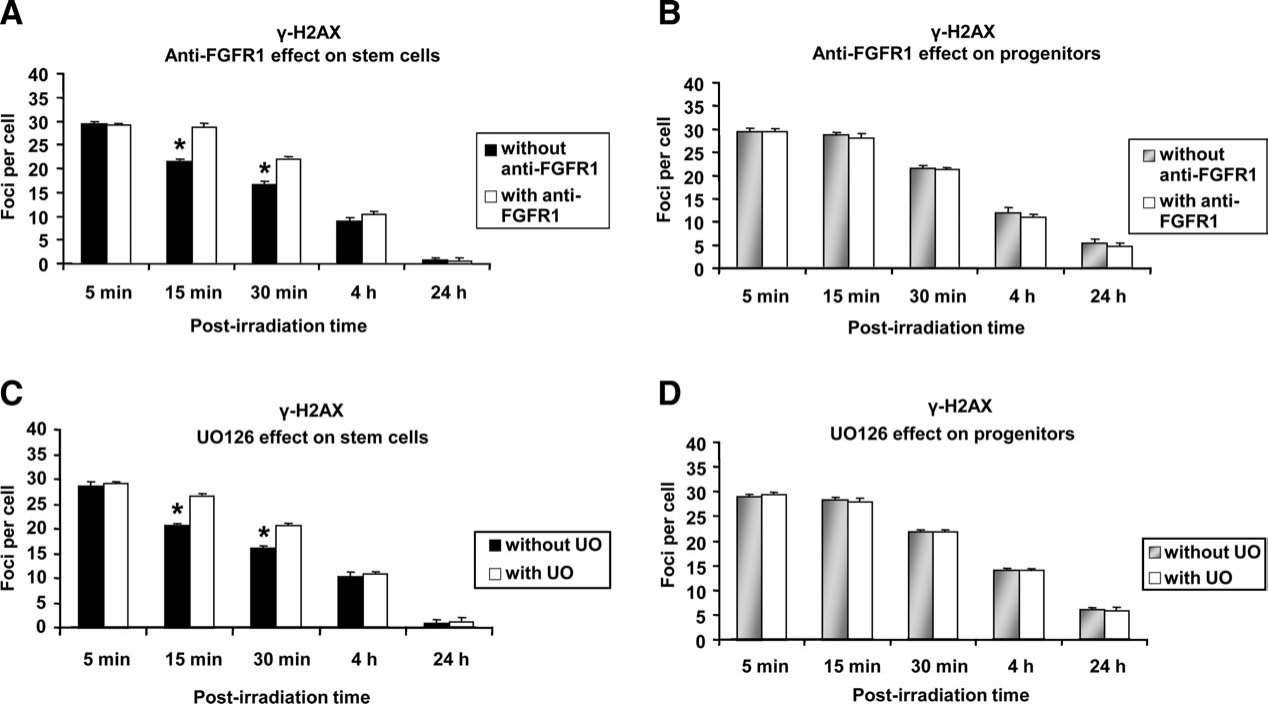
Endogenous fibroblast growth factor type 2 (FGF2) signaling regulates double-strand break repair in keratinocyte stem cells. The formation of γH2AX foci was studied in irradiated cells after blocking the FGF2 signaling pathway with an anti-FGFR1 antibody or the Ras-mitogen-activated protein kinase 1 (MAPK1) inhibitor UO126. At least three independent experiments on different skin samples were performed for each time point. Approximately 100 cells per time point were counted in each experiment. *, *p* < .01. **(A)**: Blocking the FGF2 pathway via its receptor abolished the early decrease in the number of γH2AX foci per stem cell. Incubation with a control mouse IgM isotype antibody had no effect on the number of foci (Supporting Information 3). **(B)**: No effect of antibody treatment was observed in the progenitor cells. **(C)**: Blocking the FGF2 pathway at the MAPK level via UO126 in stem cells inhibited the early decrease in the number of γH2AX foci per cell. **(D)**: No effect of UO126 treatment was observed in the progenitor cells. Abbreviations: FGFR1, fibroblast growth factor receptor 1; γH2AX, histone H2AX phosphorylated on serine 139; UO, UO126.

To assess the possible functional relationship between FGF2 signaling and double-strand break repair in keratinocyte stem cells, the γH2AX focus formation was characterized in irradiated keratinocytes following blockade of the FGF2 signaling pathway. The pathway was first inhibited at the receptor level by adding the specific FGFR1 blocking antibody 30 minutes before radiation exposure. In the stem cells, this blockade curbed the rapid decrease in the number of γH2AX foci at 15- and 30-minute postirradiation (Fig. 6A), while it had no impact on the progenitor cells (Fig. 6B). In the stem cells, the blockade had no significant effect at later time points, with similar numbers of γH2AX foci detected 4 and 24 hours after exposure irrespective of pretreatment with the blocking antibody. The FGF2 signaling pathway was then inhibited at the cytoplasm level, using the MAPK1 inhibitor UO126 30 minutes before irradiation. In the stem cells, UO126 inhibited the rapid decrease in the number of γH2AX foci 15 and 30 minutes after exposure (Fig. 6C), whereas it had no effect on the progenitors at any time point (Fig. 6D), nor on the stem cells at 4- and 24-hour postirradiation. These results showed that the early phase of DNA double-strand break repair was regulated by endogenous FGF2.

### Exogenous FGF2 Supplementation Induces Rapid DNA Double-Strand Break Repair in Keratinocyte Progenitor Cells

**Figure 7:**
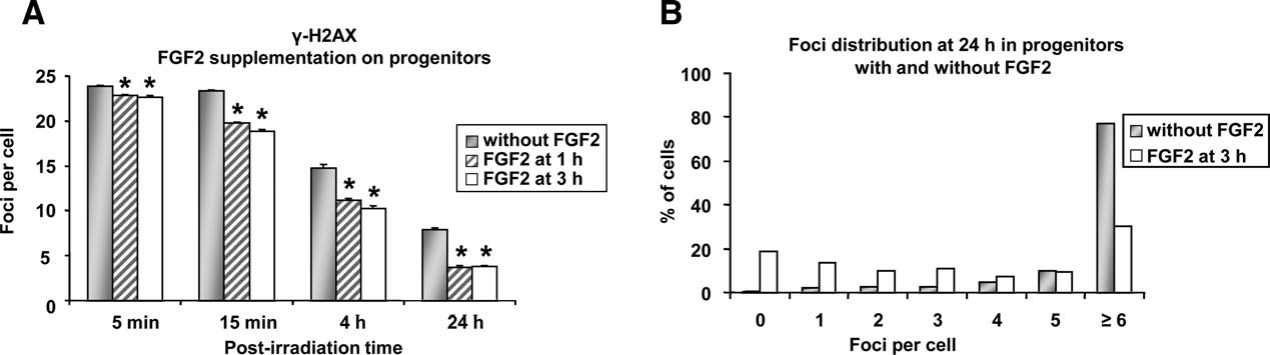
Exogenous FGF2 activates DNA double-strand break repair in keratinocyte progenitor cells. **(A)**: The FGF2 supplementation induced rapid DNA repair in the progenitor cells. Human recombinant FGF2 was added to the culture medium at 3 hours before radiation exposure. The γH2AX assay characterized double-strand break repair in irradiated progenitor cells. Five different experiments on independent skin samples were performed for each time point. A mean of 600 cells per time point was counted. *, *p* < .01. **(B)**: The distribution of the number of γH2AX foci at 24 hours after irradiation is shown for the progenitor cells, either untreated or supplemented with FGF2 at 3 hours before irradiation. The majority of untreated cells had more than four foci per nucleus (92%). After FGF2 supplementation, only 47% of progenitors presented four or more foci per nucleus. The distribution of foci in cells supplemented with FGF2 at 1 hour before irradiation is shown in Supporting Information 5. Abbreviation: FGF2, fibroblast growth factor type 2; γH2AX, histone H2AX phosphorylated on serine 139.

The progenitor cells were treated with exogenous human recombinant FGF2 at 1 or 3 hours before radiation exposure. The γH2AX foci were more rapidly lost in the treated than the untreated cells, regardless of the supplementation time (Fig. 7A). Moreover, at 24-hour postirradiation, the foci number decreased from a mean seven foci per nucleus in the untreated cells to three in the FGF2-treated cells. Consequently, the number of cells with more than four residual foci at 24 hours was reduced to 47% after exogenous FGF2 treatment, compared with 92% in untreated cells (Fig. 7B and Supporting Information Fig. 4). These results showed that exogenous FGF2 induced a faster and more efficient double-strand break repair in keratinocyte progenitor cells. They also illustrated the prosurvival action of FGF2.

## DISCUSSION

Stem cells can use various mechanisms to protect their genome and maintain genomic stability [28]. After external insult, one effective mechanism of tissue protection is the elimination of damaged cells, which can result from apoptosis, forced differentiation or premature senescence. Thus, mouse and human embryonic stem cells are hypersensitive to the toxic effects of DNA-damaging agents such as UV-C, N-meth-yl-N -nitro-N-nitrosoguanidine (MNNG), and ionizing radiation [29–32]. Tissue stem cells, however, have to handle other important functions: tissue homeostasis and regeneration after wounding. Ensuring these functions requires avoiding stem cell depletion, which can be achieved through the resistance of stem cells to external insults. A major mechanism of resistance to DNA-damaging agents is to enhance DNA repair, which can be realized at different levels of the related pathways, from cell signaling to repair activities.

We previously demonstrated that keratinocyte stem cells do not respond to the clinically relevant dose of 2 Gy by massive death induction, and that they are more radioresistant than their direct progeny, the progenitor cells [23]. The present study addressed their DNA repair potential. We first showed that global DNA repair, as measured at the individual cell level by the alkaline comet assay, was more rapid in stem cells than in progenitor cells. This alkaline comet assay measures all DNA breaks produced either by irradiation or through repair intermediates. It essentially evaluates single-strand breaks, as they are the most numerous breaks after γ-rays [25]. We then studied double-strand breaks. Of the many different classes of radiation-induced damage, DNA double-strand breaks are the most deleterious as unrepaired DNA double-strand breaks (DSBs) can result in cell death, and misrepair can cause chromosomal translocations, a possible early step of carcinogenesis [33]. The γH2AX assay indicated that double-strand break repair was more rapid and efficient in keratinocyte stem cells than in progenitor cells. The difference was extremely reproducible among keratinoctes isolated from 11 different donors. Moreover, high levels of residual unrepaired foci at 24 hours persisted only in the progenitor cells. As a general correlation has been demonstrated between cell death and the number of residual foci at 24 hours after exposure [26], the present focus-count data argue for a higher stem cell radioresistance as compared with progenitor cells.

Mammalian cells use two major pathways to repair DNA double-strand breaks: NHEJ, which is efficient in any phase of the cell cycle, and homologous recombination, which is only efficient in the S and G2 phases because it requires a homologous DNA repair template. As both cultured cell populations were mainly in the G0/G1 phase of the cell cycle up to at least 24 hours after irradiation, the NHEJ pathway is most probably the major repair pathway of DNA double-strand breaks during the time course used in the present study. Although keratinocyte stem cells rapidly repair their radiation-induced DNA damage, the fidelity of this repair is not yet known. As these cells represent the long-term reservoir for skin regeneration, and as NHEJ can be an error-prone pathway, it will be of crucial importance to determine whether long-term anomalies occurring in the stem cells, such as chromosomal aberrations or mutations, could be at the origin of carcinogenesis.

Taken together, the present data show that the basal layer of the human epidermis contains two-cell populations with different DNA repair capacities: the minor stem cell population exhibits a more rapid and efficient repair than the large progenitor population. These data are original, as the DNA repair issue, which has rarely been studied in adult stem cells, had never been characterized for human skin stem cells.

Cell signaling upstream of DNA repair is also a key regulatory step in the management of DNA lesions. Thus, WNT/β-catenin signaling was shown to mediate radiation resistance in mouse mammary progenitor cells [34]. Ataxia telangiectasia-mutated kinase is also a key player, as its derepression in *Atm*−/− mice increased crypt stem cell radiosensitivity [35]. After UV exposure, signaling proteins such as UV-damaged DNA binding protein-2 may be essential for the repair of cyclobutane pyrimidine dimers in keratinocyte stem cells and progenitor cells [36]. To explore various upstream regulators of the protective response of keratinocyte stem cells to radiation exposure, we performed gene profiling. This study revealed that a major specific response of the exposed stem cells was the activation of a network composed of cytokines and growth factors, including FGF2. For this factor, increased contents of its mRNA and protein were found in irradiated stem cells compared with progenitors. Moreover, key elements of the pathway were induced at the transcriptional level. We therefore postulated that this FGF2 response to radiation could be related to the activation of DNA repair in keratinocyte stem cells. It is known that FGF2 is a wide-spectrum survival factor, able to promote cell resistance to stress. Although the relationship between FGF2 and cell survival is well-documented, its potential role in DNA repair has only been assessed in HeLa cells. Ader [37] reported that γ-irradiated HeLa cells overexpressed an endogenous 24 kDa FGF2 isoform, which resulted in an increased activity of the DNA repair protein DNA-dependent protein kinase (DNA-PK). These cells exhibited faster double-strand break repair via NHEJ dependent on the activity of DNA-PK.

The present study addressed the possible role of endogenous FGF2 in the repair of DNA damage in keratinocyte stem cells. The pathway was blocked at the membrane level, targeting the FGFR1 receptor, or at the cytoplasm level, targeting MAPK1. Both blockades reduced DNA repair in the stem cells, showing that all of the types of damage studied (base damage and single-strand breaks in the comet assay and double-strand breaks in the γH2AX assay) were sensitive to FGF2. On the other hand, the addition of human recombinant FGF2 to the progenitor cells significantly activated DNA repair. Exogenous FGF2 induced a faster double-strand break repair and decreased the percentage of progenitor cells presenting a high level of residual γH2AX foci. As endogenous secretion of FGF2 protected the stem cells from radiation exposure, and as the addition of exogenous FGF2 protected the progenitor cells, we postulate that, in situ, the FGF2 secreted by the stem cell compartment may also act as a protector of the early progenitors directly derived from asymmetric stem cell division. Thus, both autocrine and paracrine effects of FGF2 may be key elements of the stem cell niche, participating in the protection of the whole tissue from genotoxic stress. Owing to the classic use of FGF2 addition in many stem cell growth media, we propose that, apart from its well-known role as a strong mitogen, this addition might protect stem cells and help to maintain their genomic integrity during long-term cell amplification.

## CONCLUSION

In conclusion, the present study showed for the first time that a human cell population enriched in epithelial stem cells possesses a high stress-induced capacity to repair DNA damage. Moreover, we show that FGF2 has an important role in these protective mechanisms by participating in the regulation of the DNA repair processes. However, it remains to be determined by which mechanisms FGF2 regulates DNA repair. As the repair of different types of damage was found regulated by FGF2, it can be speculated that a general mechanism such as chromatin modification or activation of antioxidant defenses might be involved.

## Disclosure of Potential Conflicts of Interest

The authors indicate no potential conflicts of interest.

## References

[1] Itoh N (2007). The Fgf families in humans, mice, and zebrafish: Their evolutional processes and roles in development, metabolism, and disease. Biol Pharm Bull.

[2] Schlessinger J (2004). Common and distinct elements in cellular signaling via EGF and FGF receptors. Science.

[3] Xu RH, Peck RM, Li DS (2005). Basic FGF and suppression of BMP signaling sustain undifferentiated proliferation of human ES cells. Nat Methods.

[4] Dvorak P, Dvorakova D, Hampl A (2006). Fibroblast growth factor signaling in embryonic and cancer stem cells. FEBS Lett.

[5] Eiselleova L, Matulka K, Kriz V (2009). A Complex role for FGF-2 in self-renewal, survival, and adhesion of human embryonic. Stem Cells.

[6] Aasen T, Raya A, Barrero MJ (2008). Efficient and rapid generation of induced pluripotent stem cells from human keratinocytes. Nat Biotechnol.

[7] Huangfu D, Osafune K, Maehr R (2008). Induction of pluripotent stem cells from primary human fibroblasts with only Oct4 and Sox2. Nat Biotechnol.

[8] Gritti A, Parati EA, Cova L (1996). Multipotential stem cells from the adult mouse brain proliferate and self-renew in response to basic fibroblast growth factor. J Neurosci.

[9] Mudo G, Bonomo A, Di Liberto V (2009). The FGF-2/FGFRs neurotrophic system promotes neurogenesis in the adult brain. J Neural Transm.

[10] Bithell A, Finch SE, Hornby MF (2008). Fibroblast growth factor 2 maintains the neurogenic capacity of embryonic neural progenitor cells in vitro but changes their neuronal subtype specification. Stem Cells.

[11] Choi SC, Kim SJ, Choi JH (2008). Fibroblast growth factor-2 and -4 promote the proliferation of bone marrow mesenchymal stem cells by the activation of the PI3K-Akt and ERK1/2 signaling pathways. Stem Cells Dev.

[12] Zaragosi LE, Ailhaud G, Dani C (2006). Autocrine fibroblast growth factor 2 signaling is critical for self-renewal of human multipotent adipose-derived stem cells. Stem Cells.

[13] Rider DA, Dombrowski C, Sawyer AA (2008). Autocrine fibroblast growth factor 2 increases the multipotentiality of human adipose-derived mesenchymal stem cells. Stem Cells.

[14] Houchen CW, George RJ, Sturmoski MA (1999). FGF-2 enhances intestinal stem cell survival and its expression is induced after radiation injury. Am J Physiol.

[15] Paris F, Fuks Z, Kang A (2001). Endothelial apoptosis as the primary lesion initiating intestinal radiation damage in mice. Science.

[16] Ganat Y, Soni S, Chacon M (2002). Chronic hypoxia up-regulates fibroblast growth factor ligands in the perinatal brain and induces fibroblast growth factor-responsive radial glial cells in the sub-ependymal zone. Neuroscience.

[17] Agasse F, Nicoleau C, Petit J (2007). Evidence for a major role of endogenous fibroblast growth factor-2 in apoptotic cortex-induced subventricular zone cell proliferation. Eur J Neurosci.

[18] Song H, Kwon K, Lim S (2005). Transfection of mesenchymal stem cells with the FGF-2 gene improves their survival under hypoxic conditions. Mol Cells.

[19] Watt FM, Lo Celso C, Silva-Vargas V (2006). Epidermal stem cells: An update. Curr Opin Genet Dev.

[20] Kaur P (2006). Interfollicular epidermal stem cells: Identification, challenges, potential. J Invest Dermatol.

[21] Li A, Simmons PJ, Kaur P (1998). Identification and isolation of candidate human keratinocyte stem cells based on cell surface phenotype. Proc Natl Acad Sci USA.

[22] Terunuma A, Kapoor V, Yee C (2007). Stem cell activity of human side population and alpha6 integrin-bright keratinocytes defined by a quantitative in vivo assay. Stem Cells.

[23] Rachidi W, Harfourche G, Lemaitre G (2007). Sensing radiosensitivity of human epidermal stem cells. Radiother Oncol.

[24] Gault N, Rigaud O, Poncy JL (2007). Biochemical alterations in human cells irradiated with alpha particles delivered by macro- or microbeams. Radiat Res.

[25] Olive PL, Banath JP (2006). The comet assay: A method to measure DNA damage in individual cells. Nat Protoc.

[26] Klokov D, MacPhail SM, Banath JP (2006). Phosphorylated histone H2AX in relation to cell survival in tumor cells and xenografts exposed to single and fractionated doses of X-rays. Radiother Oncol.

[27] Favata MF, Horiuchi KY, Manos EJ (1998). Identification of a novel inhibitor of mitogen-activated protein kinase kinase. J Biol Chem.

[28] Harfouche G, Martin MT (2010). Response of normal stem cells to ionizing radiation: A balance between homeostasis and genomic stability. Mutat Res.

[29] Corbet SW, Clarke AR, Gledhill S (1999). P53-dependent and -independent links between DNA-damage, apoptosis and mutation frequency in ES cells. Oncogene.

[30] Roos WP, Christmann M, Fraser ST (2007). Mouse embryonic stem cells are hypersensitive to apoptosis triggered by the DNA damage *O*(6)-methylguanine due to high E2F1 regulated mismatch repair. Cell Death Differ.

[31] Filion TM, Qiao M, Ghule PN (2009). Survival responses of human embryonic stem cells to DNA damage. J Cell Physiol.

[32] Hong Y, Stambrook PJ (2004). Restoration of an absent G1 arrest and protection from apoptosis in embryonic stem cells after ionizing radiation. Proc Natl Acad Sci USA.

[33] Jeggo PA, Lobrich M (2007). DNA double-strand breaks: Their cellular and clinical impact?. Oncogene.

[34] Woodward WA, Chen MS, Behbod F (2007). WNT/beta-catenin mediates radiation resistance of mouse mammary progenitor cells. Proc Natl Acad Sci USA.

[35] Ch'ang HJ, Maj JG, Paris F (2005). ATM regulates target switching to escalating doses of radiation in the intestines. Nat Med.

[36] Nijhof JG, van Pelt C, Mulder AA (2007). Epidermal stem and progenitor cells in murine epidermis accumulate UV damage despite NER proficiency. Carcinogenesis.

[37] Ader I, Muller C, Bonnet J (2002). The radioprotective effect of the 24 kDa FGF-2 isoform in HeLa cells is related to an increased expression and activity of the DNA dependent protein kinase (DNA-PK) catalytic subunit. Oncogene.

