# Supplementary figures and images for: Fibroblast Growth Factor Type 2 Signaling Is Critical for DNA Repair in Human Keratinocyte Stem Cells
Source: Stem Cells. 2010 Sep;28(9):1639–48. doi: 10.1002/stem.485 (PMC2996082; doi:10.1002/stem.485)

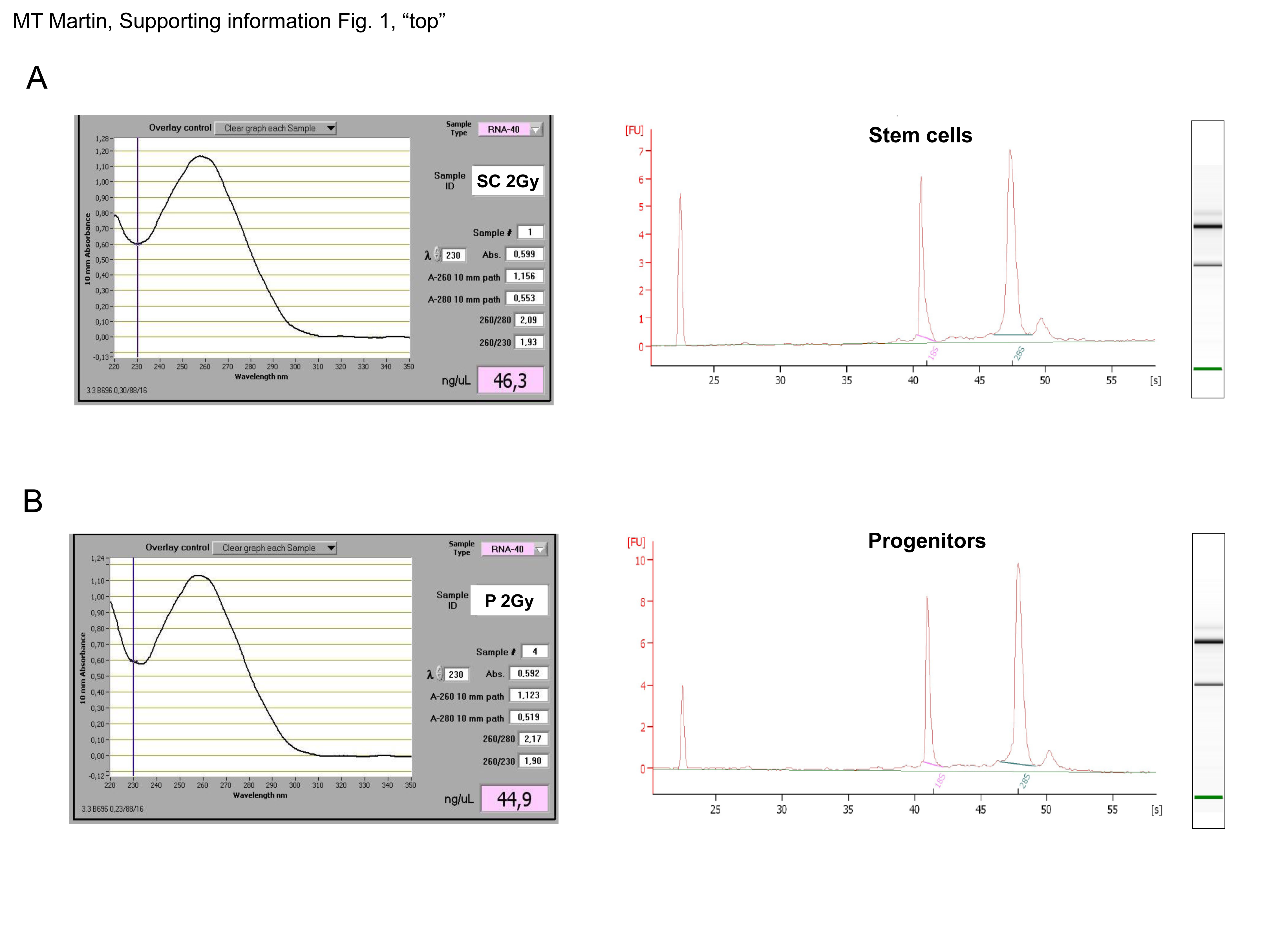

Supplement: Supplementary file 1 [file stem0028-1639-SD1.tif]

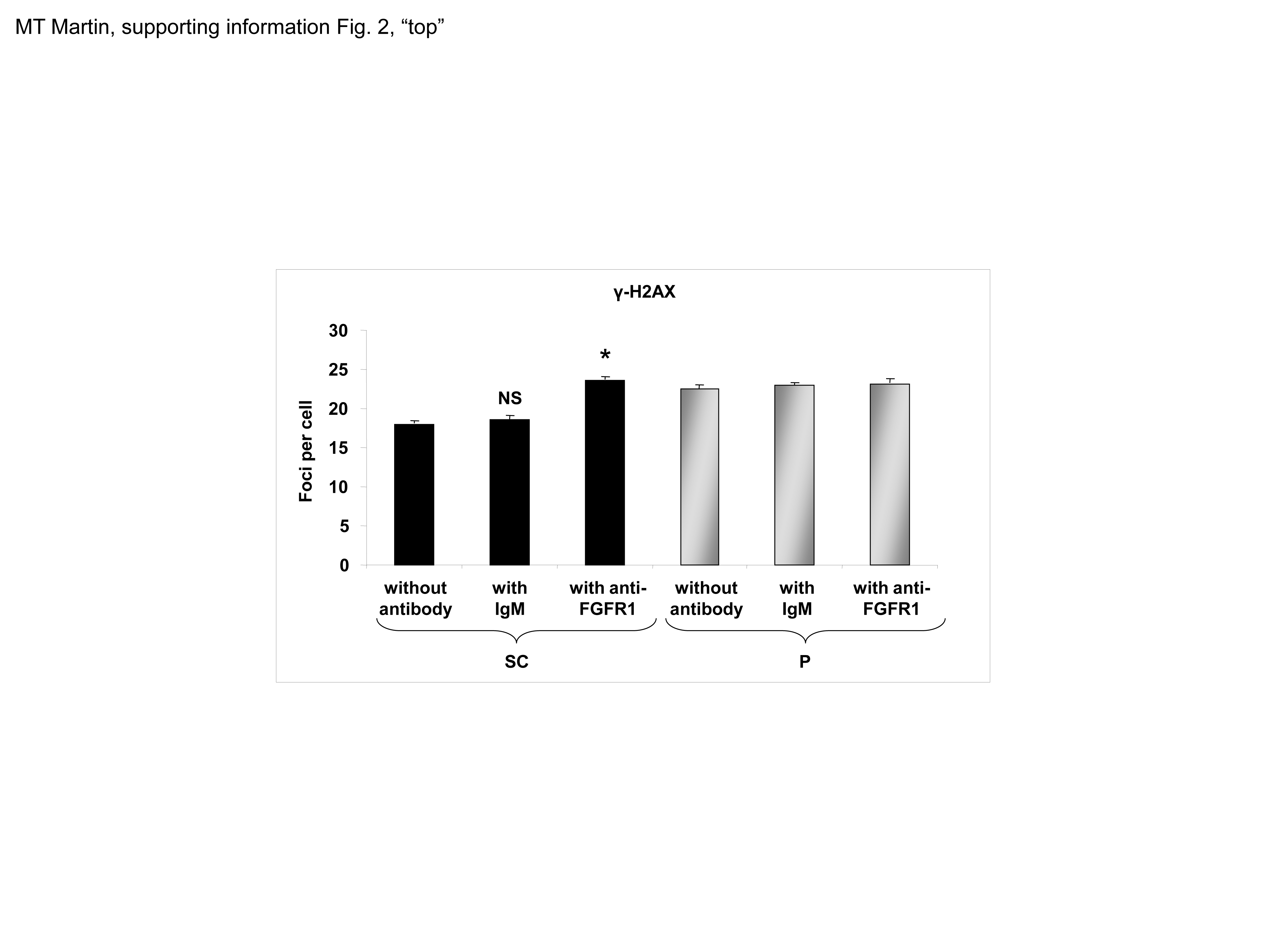

Supplement: Supplementary file 2 [file stem0028-1639-SD2.tif]

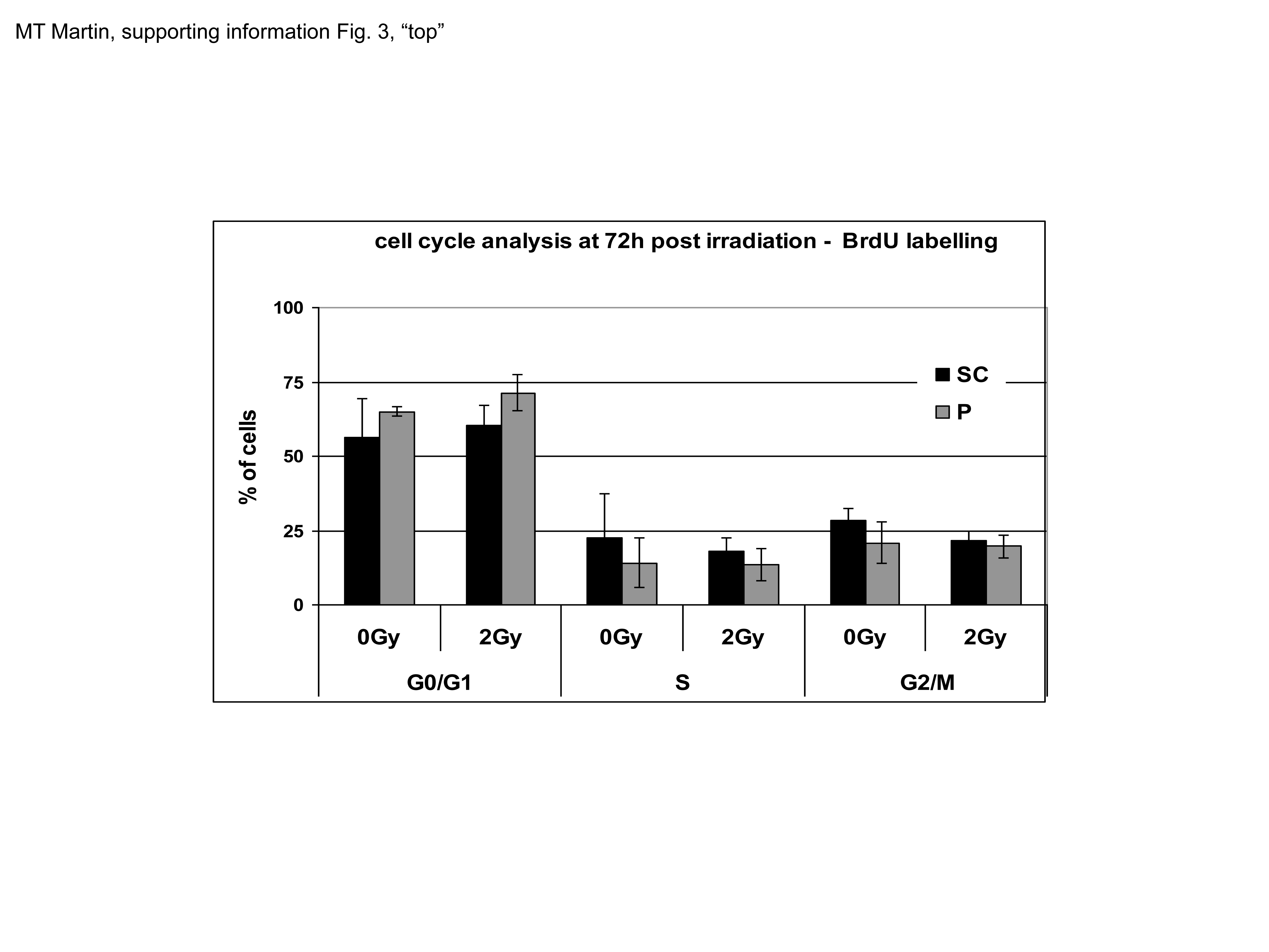

Supplement: Supplementary file 3 [file stem0028-1639-SD3.tif]

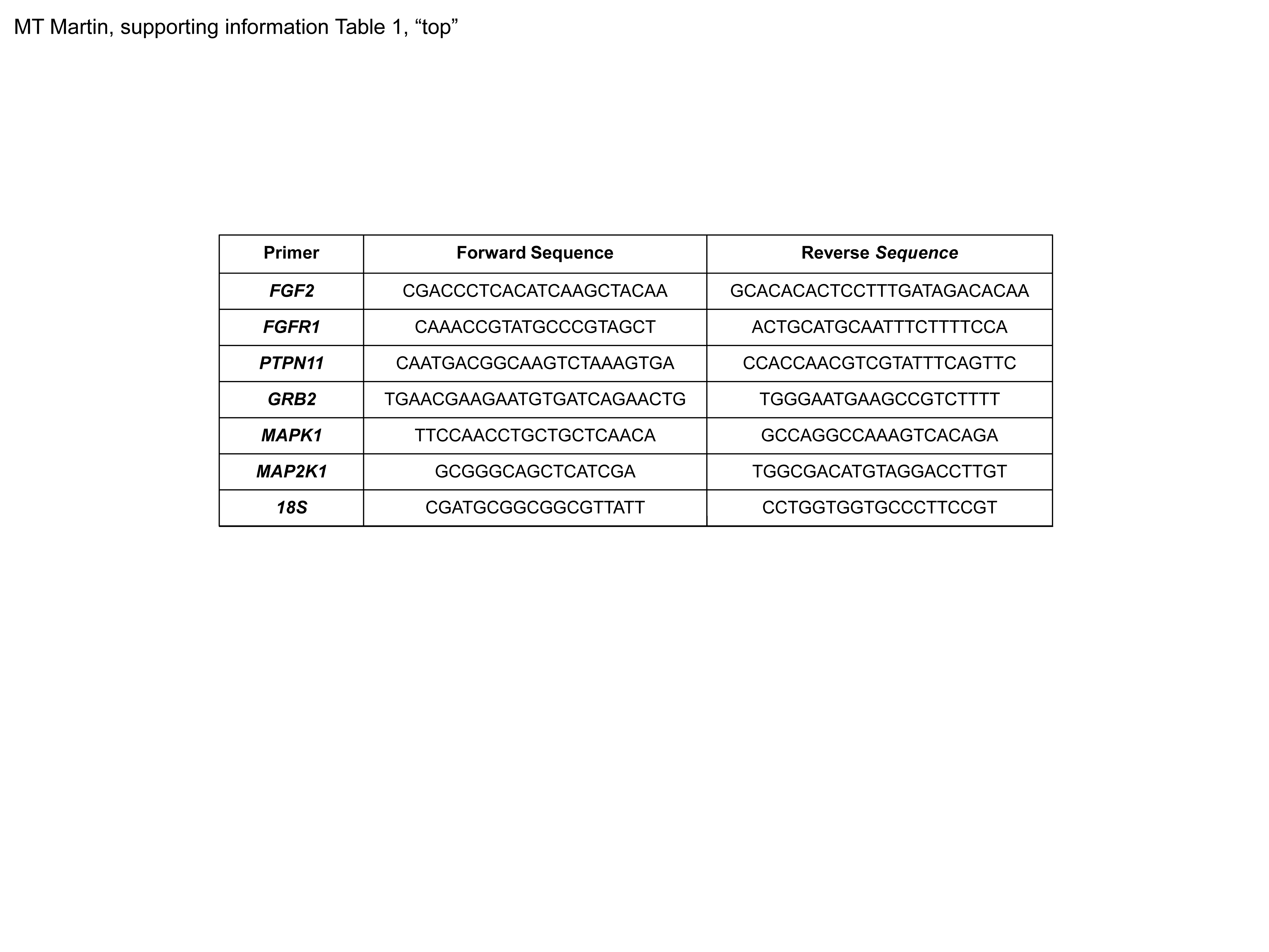

Supplement: Supplementary file 4 [file stem0028-1639-SD4.tif]
