# Supplementary material for: Fibroblast Growth Factor Type 2 Signaling Is Critical for DNA Repair in Human Keratinocyte Stem Cells
Source: Stem Cells. 2010 Sep;28(9):1639–48. doi: 10.1002/stem.485 (PMC2996082; doi:10.1002/stem.485)
Supplement: Supplementary file 5 [file stem0028-1639-SD5.doc]

| **Induced genes in irradiated keratinocyte stem cells** | | |  |
| --- | --- | --- | --- |
| **Genbank** | **Common** | **Gene description** | **Fold change** |
| NM_003246 | THBS1 | thrombospondin 1 | 13.12 |
| NM_012242 | DKK1 | dickkopf homolog 1 (Xenopus laevis) | 11.31 |
| NM_003483 | HMGA2 | high mobility group AT-hook 2 | 7.802 |
| NM_003236 | TGFA | transforming growth factor, alpha | 7.488 |
| NM_014363 | SACS | spastic ataxia of Charlevoix-Saguenay (sacsin) | 6.822 |
| NM_020182 | TMEPAI | transmembrane, prostate androgen induced RNA | 6.12 |
| NM_003979 | RAI3 | G protein-coupled receptor, family C, group 5, member A | 6.111 |
| NM_016201 | AMOTL2 | angiomotin like 2 | 5.869 |
| NM_003236 | TGFA | transforming growth factor, alpha | 5.69 |
| NM_001354 | AKR1C2 | aldo-keto reductase family 1, member C2 | 5.435 |
| NM_015685 | SDCBP2 | syndecan binding protein (syntenin) 2 | 5.28 |
| NM_019060 | C1orf42 | chromosome 1 open reading frame 42 | 5.183 |
| NM_022767 | FLJ12484 | hypothetical protein FLJ12484 | 5.138 |
| NM_138573 | NRG4 | neuregulin 4 | 5.047 |
| NM_006022 | TGFB1I4 | TSC22 domain family 1 | 5.029 |
| NM_004760 | STK17A | serine/threonine kinase 17a (apoptosis-inducing) | 5.028 |
| NM_004852 | ONECUT2 | one cut domain, family member 2 | 4.97 |
| XM_294019 | LOC345930 | similar to ECT2 protein (Epithelial cell transforming sequence 2 oncogene) | 4.908 |
| AL833395 | LOC388418 | hypothetical gene supported by AL833395 | 4.889 |
| NM_004431 | EPHA2 | EPH receptor A2 | 4.853 |
| NM_152355 | ZNF441 | zinc finger protein 441 | 4.815 |
| NM_001394 | DUSP4 | dual specificity phosphatase 4 | 4.796 |
| NM_005704 | PTPRU | protein tyrosine phosphatase, receptor type, U | 4.796 |
| NM_000584 | IL8 | interleukin 8 | 4.779 |
| NM_001748 | CAPN2 | calpain 2, (m/II) large subunit | 4.759 |
| NM_031459 | SESN2 | sestrin 2 | 4.743 |
| NM_004062 | CDH16 | cadherin 17, LI cadherin (liver-intestine) | 4.742 |
| NM_003101 | SOAT1 | sterol O-acyltransferase (acyl-Coenzyme A: cholesterol acyltransferase) 1 | 4.69 |
| NM_176081 | DNMT2 | DNA (cytosine-5-)-methyltransferase 2 | 4.624 |
| NM_006216 | SERPINE2 | serine (or cysteine) proteinase inhibitor, clade E, member 2 | 4.559 |
| NM_021209 | CARD12 | caspase recruitment domain family, member 12 | 4.553 |
| NM_017715 | ZNF3 | zinc finger protein 3 (A8-51) | 4.511 |
| NM_032718 | MGC11332 | hypothetical protein MGC11332 | 4.474 |
| AK021428 | C6orf210 | chromosome 6 open reading frame 210 | 4.457 |
| **NM_002755** | **MAP2K1** | **mitogen-activated protein kinase kinase 1** | **4.438** |
| NM_017576 | KIF27 | kinesin family member 27 | 4.419 |
| NM_004685 | MTMR6 | myotubularin-related protein 6 | 4.385 |
| AL831866 | LOC120376 | hypothetical protein LOC120376 | 4.35 |
| NM_005515 | HLXB9 | homeo box HB9 | 4.348 |
| XM_060307 | OR5BF1 | olfactory receptor, family 5, subfamily BF, member 1 | 4.329 |
| NM_004817 | TJP2 | tight junction protein 2 (zona occludens 2) | 4.298 |
| NM_007207 | DUSP10 | dual specificity phosphatase 10 | 4.291 |
| NM_000547 | TPO | thrombopoietin | 4.288 |
| NM_006636 | MTHFD2 | methylenetetrahydrofolate dehydrogenase 2, methenyltetrahydrofolate cyclohydrolase | 4.268 |
| NM_012448 | STAT5B | signal transducer and activator of transcription 5B | 4.25 |
| NM_138720 | HIST1H2BD | histone 1, H2bd | 4.237 |
| NM_016639 | TNFRSF12A | tumor necrosis factor receptor superfamily, member 12A | 4.235 |
| NM_000020 | ACVRL1 | activin A receptor type II-like 1 | 4.206 |
| BC035696 | MGC57827 | Similar to RIKEN cDNA 2700049P18 gene | 4.196 |
| NM_006072 | CCL26 | chemokine (C-C motif) ligand 26 | 4.195 |
| AA683321 | PAR1 | coagulation factor II (thrombin) receptor | 4.172 |
| NM_002752 | MAPK9 | mitogen-activated protein kinase 9 | 4.162 |
| L26245 | BIRC5 | baculoviral IAP repeat-containing 5 (survivin) | 4.145 |
| NM_007049 | BTN2A1 | butyrophilin, subfamily 2, member A1 | 4.126 |
| NM_021006 | MGC12815 | chemokine (C-C motif) ligand 3-like 1 | 4.119 |
| NM_030970 | MGC3771 | hypothetical protein MGC3771 | 4.117 |
| NM_001717 | BNC1 | amiloride-sensitive cation channel 1, neuronal (degenerin) | 4.098 |
| NM_012334 | MYO10 | myosin X | 4.086 |
| AK022384 | FLJ34870 | FLJ34870 protein | 4.057 |
| NM_032858 | FLJ14904 | hypothetical protein FLJ14904 | 4.03 |
| NM_002268 | KPNA4 | karyopherin alpha 4 (importin alpha 3) | 4.027 |
| NM_147198 | WFDC9 | WAP four-disulfide core domain 9 | 4.026 |
| NM_032325 | MGC11102 | hypothetical protein MGC11102 | 3.996 |
| NM_002539 | ODC1 | solute carrier family 25 (mitochondrial oxodicarboxylate carrier), member 21 | 3.97 |
| NM_017527 | LY6K | lymphocyte antigen 6 complex, locus K | 3.944 |
| NM_018699 | PRDM5 | PR domain containing 5 | 3.906 |
| NM_147130 | NCR3 | natural cytotoxicity triggering receptor 3 | 3.887 |
| NM_020124 | IFNK | interferon, kappa | 3.88 |
| NM_004876 | ZNF539 | zinc finger protein 539 | 3.859 |
| NM_000270 | NP | kallikrein 8 (neuropsin/ovasin) | 3.857 |
| NM_005409 | CXCL11 | chemokine (C-X-C motif) ligand 11 | 3.853 |
| XM_038788 | TGFBR1 | transforming growth factor, beta receptor I (activin A receptor type II-like kinase, 53kDa) | 3.804 |
| NM_006449 | CDC42EP3 | CDC42 effector protein (Rho GTPase binding) 3 | 3.798 |
| NM_058172 | ANTXR2 | anthrax toxin receptor 2 | 3.798 |
| NM_016561 | BFAR | bifunctional apoptosis regulator | 3.784 |
| AL365373 | C19orf10 | chromosome 19 open reading frame 10 | 3.777 |
| BC031867 | KIAA1967 | KIAA1967 | 3.761 |
| NM_004271 | LY86 | lymphocyte antigen 86 | 3.737 |
| NM_000557 | GDF5 | growth differentiation factor 5 (cartilage-derived morphogenetic protein-1) | 3.732 |
| NM_012124 | CHORDC1 | cysteine and histidine-rich domain (CHORD)-containing, zinc binding protein 1 | 3.721 |
| HSHTPRH07 | OR2L2 | olfactory receptor, family 2, subfamily L, member 2 | 3.714 |
| NM_005729 | PPIF | peptidylprolyl isomerase F (cyclophilin F) | 3.682 |
| NM_138799 | OACT2 | O-acyltransferase (membrane bound) domain containing 2 | 3.68 |
| AL357212 | GPR133 | G protein-coupled receptor 133 | 3.669 |
| AB062484 | CALD1 | caldesmon 1 | 3.664 |
| NM_018092 | NETO2 | neuropilin (NRP) and tolloid (TLL)-like 2 | 3.655 |
| NM_012250 | RRAS2 | related RAS viral (r-ras) oncogene homolog 2 | 3.641 |
| NM_133328 | DEDD2 | death effector domain containing 2 | 3.637 |
| NM_025195 | TRIB1 | tribbles homolog 1 (Drosophila) | 3.633 |
| NM_031968 | NARF | nuclear prelamin A recognition factor | 3.622 |
| AF147404 | FALZ | fetal Alzheimer antigen | 3.596 |
| NM_033258 | GNG8 | guanine nucleotide-binding protein (G protein), gamma-transducing activity polypeptide 2 | 3.542 |
| AK094369 | IGSF4 | immunoglobulin superfamily, member 4 | 3.538 |
| AK057533 | RBPMS | RNA-binding protein with multiple splicing | 3.532 |
| NM_012341 | GTPBP4 | GTP-binding protein 4 | 3.503 |
| NM_024524 | AFURS1 | ATPase type 13A3 | 3.501 |
| BF125408 | FNBP3 | formin-binding protein 3 | 3.492 |
| BC023657 | LOC284804 | hypothetical protein LOC284804 | 3.488 |
| NM_014670 | BZW1 | basic leucine zipper and W2 domains 1 | 3.467 |
| NM_032730 | RTN4IP1 | reticulon 4 interacting protein 1 | 3.454 |
| NM_000903 | NQO1 | NAD(P)H dehydrogenase, quinone 1 | 3.451 |
| NM_001274 | CHEK1 | CHK1 checkpoint homolog (S. pombe) | 3.413 |
| NM_005292 | GPR18 | G protein-coupled receptor 18 | 3.395 |
| AK025332 | PLXNA2 | plexin A2 | 3.393 |
| NM_078467 | CDKN1A | p21 (CDKN1A)-activated kinase 3 | 3.39 |
| NM_033661 | WDR4 | WD repeat domain 4 | 3.385 |
| AA157955 | SC4MOL | sterol-C4-methyl oxidase-like | 3.307 |
| NM_006000 | TUBA1 | tubulin, alpha 1 (testis specific) | 3.306 |
| NM_004752 | GCM2 | glial cells missing homolog 2 (Drosophila) | 3.282 |
| NM_017816 | LYAR | hypothetical protein FLJ20425 | 3.27 |
| NM_020127 | TUFT1 | tuftelin 1 | 3.269 |
| NM_007178 | STRAP | serum response factor binding protein 1 | 3.258 |
| NM_002160 | TNC | troponin C, slow | 3.256 |
| NM_014302 | SEC61G | Sec61 gamma subunit | 3.247 |
| NM_020177 | FEM1C | fem-1 homolog c (C. elegans) | 3.234 |
| NM_006367 | CAP1 | CAP, adenylate cyclase-associated protein 1 (yeast) | 3.223 |
| NM_022578 | CSH2 | chorionic somatomammotropin hormone 2 | 3.197 |
| NM_032589 | DSCR8 | Down syndrome critical region gene 8 | 3.185 |
| NM_014670 | BZW1 | basic leucine zipper and W2 domains 1 | 3.169 |
| NM_152284 | Shax3 | chromatin modifying protein 4C | 3.153 |
| NM_005338 | HIP1 | huntingtin interacting protein 1 | 3.14 |
| NM_015420 | DKFZP564O0463 | gene model 83 | 3.138 |
| NM_018509 | PRO1855 | hypothetical protein PRO1855 | 3.123 |
| NM_000275 | OCA2 | oculocutaneous albinism II (pink-eye dilution homolog, mouse) | 3.119 |
| NM_001945 | DTR | heparin-binding EGF-like growth factor | 3.116 |
| NM_002354 | TACSTD1 | tumor-associated calcium signal transducer 1 | 3.111 |
| NM_002627 | PFKP | phosphofructokinase, platelet | 3.107 |
| NM_001900 | CST5 | cystatin D | 3.091 |
| **NM_002086** | **GRB2** | **growth factor receptor-bound protein 2** | **3.091** |
| NM_021249 | SNX6 | sorting nexin 6 | 3.075 |
| NM_001860 | SLC31A2 | solute carrier family 31 (copper transporters), member 2 | 3.06 |
| NM_002970 | SAT | spermidine/spermine N1-acetyltransferase | 3.048 |
| NM_005760 | CEBPZ | DNA-damage-inducible transcript 3 | 3.04 |
| NM_003191 | TARS | threonyl-tRNA synthetase | 3.028 |
| AB037820 | MARCH-IV | membrane-associated ring finger (C3HC4) 4 | 3.024 |
| NM_018465 | C9orf46 | chromosome 9 open reading frame 46 | 3.024 |
| BC012486 | KRTAP2-4 | keratin associated protein 2-4 | 3.015 |
| BC045765 | SHANK3 | SH3 and multiple ankyrin repeat domains 3 | 3.01 |
| NM_002574 | PRDX1 | peroxiredoxin 1 | 3.008 |
| NM_003114 | SPAG1 | sperm associated antigen 1 | 2.997 |
| NM_018107 | RBM23 | RNA binding motif protein 23 | 2.989 |
| NM_017755 | FLJ20303 | NOL1/NOP2/Sun domain family, member 2 | 2.981 |
| NM_004782 | SNAP29 | synaptosomal-associated protein, 29kDa | 2.979 |
| NM_007350 | PHLDA1 | pleckstrin homology-like domain, family A, member 1 | 2.966 |
| BC029799 | DMRTC1 | DMRT-like family C1 | 2.96 |
| NM_005112 | WDR1 | WD repeat domain 1 | 2.94 |
| NM_016584 | IL23A | interleukin 23, alpha subunit p19 | 2.918 |
| XM_113743 | TMEM16F | transmembrane protein 16F | 2.913 |
| **NM_000604** | **FGFR1** | **fibroblast growth factor receptor 1 (fms-related tyrosine kinase 2, Pfeiffer syndrome)** | **2.901** |
| NM_006795 | EHD1 | EH-domain containing 1 | 2.897 |
| NM_153344 | C6orf141 | chromosome 6 open reading frame 141 | 2.896 |
| NM_015444 | RIS1 | ubiquinol-cytochrome c reductase, Rieske iron-sulfur polypeptide 1 | 2.863 |
| NM_001347 | DGKQ | diacylglycerol kinase, theta 110kDa | 2.861 |
| AK021634 | POLR2B | polymerase (RNA) II (DNA directed) polypeptide B, 140kDa | 2.858 |
| NM_001393 | ECM2 | extracellular matrix protein 2, female organ and adipocyte specific | 2.854 |
| NM_016306 | DNAJB11 | DnaJ (Hsp40) homolog, subfamily B, member 11 | 2.849 |
| NM_018983 | NOLA1 | nucleolar protein family A, member 1 (H/ACA small nucleolar RNPs) | 2.826 |
| AK090441 | FLJ46041 | FLJ46041 protein | 2.824 |
| AK094373 | ADARB2 | adenosine deaminase, RNA-specific, B2 (RED2 homolog rat) | 2.823 |
| NM_005648 | TCEB1 | transcription elongation factor B (SIII), polypeptide 1 (15kDa, elongin C) | 2.822 |
| NM_023938 | SARG | specifically androgen-regulated protein | 2.817 |
| NM_003971 | SPAG9 | sperm associated antigen 9 | 2.808 |
| NM_004735 | LRRFIP1 | leucine-rich repeat (in FLII) interacting protein 1 | 2.804 |
| NM_002844 | PTPRK | protein tyrosine phosphatase, receptor type, K | 2.784 |
| BC009524 | PSMD14 | proteasome (prosome, macropain) 26S subunit, non-ATPase, 14 | 2.771 |
| AK023045 | LOC340351 | hypothetical protein LOC340351 | 2.768 |
| NM_003937 | KYNU | kynureninase (L-kynurenine hydrolase) | 2.766 |
| NM_005721 | ACTR3 | ARP3 actin-related protein 3 homolog (yeast) | 2.76 |
| NM_032735 | MGC13168 | hypothetical protein MGC13168 | 2.742 |
| NM_015641 | TES | testis-derived transcript (3 LIM domains) | 2.718 |
| NM_021149 | COTL1 | coactosin-like 1 (Dictyostelium) | 2.708 |
| NM_002897 | RBMS1 | RNA-binding motif, single-stranded interacting protein 1 | 2.704 |
| NM_004846 | EIF4EL3 | eukaryotic translation initiation factor 4E member 2 | 2.701 |
| BC033490 | LOC285016 | hypothetical protein LOC285016 | 2.685 |
| NM_003681 | PDXK | pyridoxal (pyridoxine, vitamin B6) kinase | 2.681 |
| NM_006339 | HMG20B | high-mobility group 20B | 2.674 |
| **NM_002006** | **FGF2** | **fibroblast growth factor 2** | **2.671** |
| NM_017723 | FLJ20245 | hypothetical protein FLJ20245 | 2.665 |
| NM_016038 | SBDS | Shwachman-Bodian-Diamond syndrome | 2.663 |
| NM_024331 | C20orf121 | chromosome 20 open reading frame 121 | 2.658 |
| NM_080725 | C20orf139 | chromosome 20 open reading frame 139 | 2.657 |
| NM_006580 | CLDN16 | claudin 16 | 2.647 |
| NM_015070 | KIAA0853 | KIAA0853 | 2.641 |
| NM_004805 | POLR2D | polymerase (RNA) II (DNA directed) polypeptide D | 2.623 |
| NM_024115 | SARG | specifically androgen-regulated protein | 2.612 |
| NM_170610 | HIST1H2BA | histone 1, H2ba | 2.612 |
| NM_080927 | DCBLD2 | discoidin, CUB and LCCL domain containing 2 | 2.611 |
| NM_001295 | CCR1 | chemokine (C-C motif) receptor 1 | 2.608 |
| NM_018530 | GSDML | gasdermin-like | 2.601 |
| NM_172390 | NFATC1 | nuclear factor of activated T-cells, cytoplasmic, calcineurin-dependent 1 | 2.601 |
| NM_032825 | ZNF382 | zinc finger protein 382 | 2.545 |
| NM_015458 | MTMR9 | myotubularin related protein 9 | 2.536 |
| NM_000596 | IGFBP1 | insulin-like growth factor binding protein 1 | 2.534 |
| NM_016146 | TRAPPC4 | trafficking protein particle complex 4 | 2.523 |
| NM_004194 | ADAM22 | a disintegrin and metalloproteinase domain 22 | 2.523 |
| NM_004179 | TPH1 | tryptophan hydroxylase 1 (tryptophan 5-monooxygenase) | 2.522 |
| NM_032175 | FLJ12787 | Src-associated protein SAW | 2.504 |
| NM_173080 | SPRR4 | small proline-rich protein 4 | 2.503 |
| XM_038933 | USP24 | ubiquitin-specific protease 24 | 2.5 |
| NM_031480 | RIOK1 | RIO kinase 1 (yeast) | 2.496 |
| NM_001949 | E2F3 | E2F transcription factor 3 | 2.494 |
| NM_014325 | CORO1C | coronin, actin binding protein, 1C | 2.487 |
| NM_022366 | TFB2M | transcription factor B2, mitochondrial | 2.482 |
| NM_178537 | Beta4GalNAc-T4 | beta1,4-N-acetylgalactosaminyltransferases IV | 2.478 |
| NM_016525 | UBAP1 | ubiquitin associated protein 1 | 2.473 |
| NM_153341 | IBRDC3 | IBR domain containing 3 | 2.468 |
| NM_022130 | GOLPH3 | golgi phosphoprotein 3 (coat-protein) | 2.466 |
| NM_003090 | SNRPA1 | small nuclear ribonucleoprotein polypeptide A' | 2.464 |
| NM_001450 | FHL2 | four and a half LIM domains 2 | 2.461 |
| NM_004728 | DDX21 | DEAD (Asp-Glu-Ala-Asp) box polypeptide 56 | 2.457 |
| NM_006773 | DDX18 | DEAD (Asp-Glu-Ala-Asp) box polypeptide 18 | 2.451 |
| NM_002808 | PSMD2 | proteasome (prosome, macropain) 26S subunit, non-ATPase, 2 | 2.442 |
| NM_014851 | KIAA0469 | kelch-like 21 (Drosophila) | 2.442 |
| AK022337 | NEBL | nebulette | 2.439 |
| NM_000606 | C8G | complement component 8, gamma polypeptide | 2.43 |
| XM_353037 | LOC387885 | hypothetical LOC387885 | 2.428 |
| AK091508 | LOC441421 | hypothetical gene supported by AK091508 | 2.426 |
| NM_031445 | MGC4268 | hypothetical protein MGC4268 | 2.422 |
| NM_006328 | RBM14 | RNA binding motif protein 14 | 2.41 |
| NM_000716 | C4BPB | complement component 4 binding protein, beta | 2.408 |
| AK024480 | LOC126917 | hypothetical protein LOC126917 | 2.4 |
| NM_003463 | PTP4A1 | protein tyrosine phosphatase type IVA, member 1 | 2.399 |
| **NM_032904** | **PTPN11** | **protein tyrosine phosphatase, non-receptor type 11 (Noonan syndrome 1)** | **2.398** |
| NM_006170 | NOL1 | nucleolar protein 1, 120kDa | 2.397 |
| NM_015238 | KIBRA | KIBRA protein | 2.394 |
| NM_000529 | MC2R | melanocortin 2 receptor (adrenocorticotropic hormone) | 2.378 |
| NM_004124 | GMFB | glia maturation factor, beta | 2.376 |
| NM_004492 | GTF2A2 | general transcription factor IIA, 2, 12kDa | 2.364 |
| NM_001827 | CKS2 | CDC28 protein kinase regulatory subunit 2 | 2.363 |
| NM_006541 | TXNL2 | thioredoxin-like 2 | 2.359 |
| NM_138572 | TBN | taube nuss homolog (mouse) | 2.352 |
| NM_015646 | RAP1B | RAP1B, member of RAS oncogene family | 2.351 |
| NM_175737 | LOC152831 | klotho beta like | 2.348 |
| NM_006938 | SNRPD1 | small nuclear ribonucleoprotein D2 polypeptide 16.5kDa | 2.346 |
| NM_175878 | MGC57211 | hypothetical protein MGC57211 | 2.346 |
| NM_032704 | TUBA6 | tubulin alpha 6 | 2.339 |
| NM_014745 | LOC348180 | hypothetical protein LOC348180 | 2.333 |
| NM_017866 | FLJ20533 | hypothetical protein FLJ20533 | 2.327 |
| NM_003201 | TFAM | transcription factor A, mitochondrial | 2.323 |
| NM_005765 | ATP6AP2 | ATPase, H+ transporting, lysosomal accessory protein 2 | 2.32 |
| NM_007173 | PRSS23 | protease, serine, 23 | 2.314 |
| NM_005857 | ZMPSTE24 | zinc metallopeptidase (STE24 homolog, yeast) | 2.314 |
| NM_177966 | 2'-PDE | 2'-phosphodiesterase | 2.303 |
| NM_032338 | MGC14817 | hypothetical protein MGC14817 | 2.3 |
| **NM_016567** | **BCCIP** | **BRCA2 and CDKN1A interacting protein** | **2.299** |
| NM_015932 | C13orf12 | chromosome 13 open reading frame 12 | 2.298 |
| NM_003879 | CFLAR | CASP8 and FADD-like apoptosis regulator | 2.292 |
| NM_003340 | UBE2D3 | ubiquitin-conjugating enzyme E2D 3 (UBC4/5 homolog, yeast) | 2.292 |
| NM_015508 | TIPARP | TCDD-inducible poly(ADP-ribose) polymerase | 2.291 |
| NM_000317 | PTS | 6-pyruvoyltetrahydropterin synthase | 2.29 |
| NM_012479 | YWHAG | tyrosine 3-monooxygenase/tryptophan 5-monooxygenase activation protein, gamma polypeptide | 2.286 |
| NM_020230 | PPAN | peter pan homolog (Drosophila) | 2.282 |
| NM_003160 | AURKC | aurora kinase C | 2.28 |
| NM_006431 | CCT2 | chaperonin containing TCP1, subunit 2 (beta) | 2.276 |
| NM_016485 | C6orf55 | chromosome 6 open reading frame 55 | 2.262 |
| AY007113 | AMMECR1 | Alport syndrome, mental retardation, midface hypoplasia and elliptocytosis chromosomal region, gene 1 | 2.259 |
| NM_003610 | RAE1 | RAE1 RNA export 1 homolog (S. pombe) | 2.258 |
| NM_003350 | UBE2V2 | ubiquitin-conjugating enzyme E2 variant 2 | 2.254 |
| NM_033274 | ADAM19 | a disintegrin and metalloproteinase domain 19 (meltrin beta) | 2.252 |
| NM_153649 | TPM3 | tropomyosin 3 | 2.252 |
| NM_006762 | LAPTM5 | lysosomal associated multispanning membrane protein 5 | 2.25 |
| NM_005614 | RHEB | Ras-homolog enriched in brain pseudogene 1 | 2.248 |
| NM_176871 | PDLIM2 | PDZ and LIM domain 2 (mystique) | 2.233 |
| NM_006002 | UCHL3 | ubiquitin carboxyl-terminal esterase L3 (ubiquitin thiolesterase) | 2.229 |
| NM_001939 | DRP2 | dihydropyrimidinase-like 2 | 2.221 |
| NM_020375 | C12orf5 | chromosome 12 open reading frame 5 | 2.221 |
| NM_016040 | TMED5 | transmembrane emp24 protein transport domain containing 5 | 2.22 |
| XM_043885 | DKFZP564F0522 | DKFZP564F0522 protein | 2.217 |
| NM_007235 | XPOT | exportin, tRNA (nuclear export receptor for tRNAs) | 2.214 |
| NM_015440 | FTHFSDC1 | methylenetetrahydrofolate dehydrogenase (NADP+ dependent) 1-like | 2.212 |
| NM_016072 | CGI-141 | golgi transport 1 homolog B (S. cerevisiae) | 2.199 |
| NM_001956 | EDN2 | endothelin 2 | 2.194 |
| NM_012141 | DDX26 | DEAD/H (Asp-Glu-Ala-Asp/His) box polypeptide 26 | 2.177 |
| NM_003714 | STC2 | stanniocalcin 2 | 2.177 |
| NM_018266 | FLJ10902 | transmembrane protein 39A | 2.177 |
| NM_001729 | BTC | betacellulin | 2.177 |
| NM_006806 | BTG3 | BTG family, member 3 | 2.175 |
| NM_002748 | MAPK6 | mitogen-activated protein kinase 6 | 2.166 |
| NM_015959 | TMX2 | thioredoxin-related transmembrane protein 2 | 2.163 |
| BC035314 | BXDC1 | brix domain containing 1 | 2.161 |
| NM_030674 | SLC38A1 | solute carrier family 38, member 1 | 2.159 |
| XM_351869 | C9orf111 | chromosome 9 open reading frame 111 | 2.155 |
| NM_017819 | RG9MTD1 | RNA (guanine-9-) methyltransferase domain containing 1 | 2.153 |
| HSM804727 | UBE2D3 | ubiquitin-conjugating enzyme E2D 3 (UBC4/5 homolog, yeast) | 2.153 |
| M31157 | PTHLH | parathyroid hormone-like hormone | 2.146 |
| NM_014462 | LSM1 | LSM1 homolog, U6 small nuclear RNA associated (S. cerevisiae) | 2.145 |
| NM_001416 | EIF4A1 | eukaryotic translation initiation factor 4A, isoform 1 | 2.14 |
| NM_152132 | PSMA3 | proteasome (prosome, macropain) subunit, alpha type, 3 | 2.137 |
| NM_005479 | FRAT1 | frequently rearranged in advanced T-cell lymphomas | 2.137 |
| NM_017510 | HSGP25L2G | transmembrane emp24 protein transport domain containing 9 | 2.134 |
| AK091649 | TEX27 | testis expressed sequence 27 | 2.131 |
| NM_181528 | NAT5 | N-acetyltransferase 5 (ARD1 homolog, S. cerevisiae) | 2.13 |
| NM_032017 | MGC4796 | Ser/Thr-like kinase | 2.128 |
| NM_005038 | PPID | peptidylprolyl isomerase D (cyclophilin D) | 2.127 |
| NM_002037 | FYN | FYN oncogene related to SRC, FGR, YES | 2.121 |
| NM_152484 | ZNF569 | zinc finger protein 569 | 2.118 |
| NM_012307 | EPB41L3 | erythrocyte membrane protein band 4.1-like 3 | 2.117 |
| NM_020368 | SAS10 | disrupter of silencing 10 | 2.109 |
| NM_014671 | UBE3C | ubiquitin protein ligase E3C | 2.108 |
| NM_001844 | COL2A1 | collagen, type II, alpha 1 (primary osteoarthritis, spondyloepiphyseal dysplasia, congenital) | 2.108 |
| AK094155 | UBE2J2 | ubiquitin-conjugating enzyme E2, J2 (UBC6 homolog, yeast) | 2.106 |
| NM_006097 | MYL9 | myosin, light polypeptide 9, regulatory | 2.103 |
| NM_025085 | TBDN100 | NMDA receptor regulated 1 | 2.1 |
| NM_024596 | MCPH1 | microcephaly, primary autosomal recessive 1 | 2.098 |
| NM_080738 | EDARADD | EDAR-associated death domain | 2.097 |
| NM_033412 | MCART1 | mitochondrial carrier triple repeat 1 | 2.097 |
| NM_002788 | PSMA3 | proteasome (prosome, macropain) subunit, alpha type, 3 | 2.096 |
| NM_000265 | NCF1 | neutrophil cytosolic factor 1 (47kDa, chronic granulomatous disease, autosomal 1) | 2.096 |
| NM_015324 | KIAA0409 | KIAA0409 protein | 2.091 |
| NM_001539 | DNAJA1 | DnaJ (Hsp40) homolog, subfamily A, member 1 | 2.088 |
| D26067 | TMEM41B | transmembrane protein 41B | 2.084 |
| NM_006555 | YKT6 | SNARE protein Ykt6 | 2.083 |
| NM_177452 | TRAPPC6B | trafficking protein particle complex 6B | 2.08 |
| NM_005690 | DNM1L | dynamin 1-like | 2.08 |
| XM_117451 | LOC402617 | hypothetical LOC402617 | 2.076 |
| NM_023927 | NS3TP2 | HCV NS3-transactivated protein 2 | 2.076 |
| NM_006174 | NPY5R | neuropeptide Y receptor Y5 | 2.071 |
| NM_006149 | LGALS4 | lectin, galactoside-binding, soluble, 4 (galectin 4) | 2.068 |
| NM_006764 | IFRD2 | interferon-related developmental regulator 2 | 2.066 |
| NM_152132 | PSMA3 | proteasome (prosome, macropain) subunit, alpha type, 3 | 2.064 |
| AF285120 | MRPL47 | mitochondrial ribosomal protein L47 | 2.06 |
| NM_014999 | RAB21 | RAB21, member RAS oncogene family | 2.06 |
| NM_005860 | FSTL3 | follistatin-like 3 (secreted glycoprotein) | 2.056 |
| NM_024329 | EFHD2 | EF hand domain family, member D2 | 2.054 |
| NM_021203 | SRPRB | signal recognition particle receptor, B subunit | 2.053 |
| NM_032047 | B3GNT5 | UDP-GlcNAc:betaGal beta-1,3-N-acetylglucosaminyltransferase 5 | 2.051 |
| NM_006912 | RIT1 | B-cell CLL/lymphoma 11B (zinc finger protein) | 2.041 |
| NM_000247 | MICA | MHC class I polypeptide-related sequence A | 2.037 |
| NM_007126 | VCP | valosin-containing protein | 2.034 |
| NM_001751 | CARS | cysteinyl-tRNA synthetase | 2.027 |
| NM_005264 | GFRA1 | GDNF family receptor alpha 1 | 2.025 |
| NM_003904 | ZNF259 | zinc finger protein 259 | 2.019 |
| NM_145804 | ABTB2 | ankyrin repeat and BTB (POZ) domain containing 2 | 2.017 |
| NM_018840 | C20orf24 | chromosome 20 open reading frame 24 | 2.016 |
| NM_006948 | STCH | stress 70 protein chaperone, microsome-associated, 60kDa | 2.015 |
| NM_023934 | FUNDC2 | FUN14 domain containing 2 | 2.009 |
| NM_016243 | NQO3A2 | NAD(P)H:quinone oxidoreductase type 3, polypeptide A2 | 2.006 |
| NM_012330 | MYST4 | MYST histone acetyltransferase (monocytic leukemia) 4 | 2.006 |
| NM_002793 | PSMB1 | proteasome (prosome, macropain) subunit, beta type, 1 | 1.998 |
| NM_004360 | CDH1 | fizzy/cell division cycle 20 related 1 (Drosophila) | 1.997 |
| NM_001968 | EIF4E | eukaryotic translation initiation factor 4E | 1.994 |
| NM_005544 | IRS1 | insulin receptor substrate 1 | 1.987 |
| NM_001966 | EHHADH | enoyl-Coenzyme A, hydratase/3-hydroxyacyl Coenzyme A dehydrogenase | 1.986 |
| NM_139313 | YME1L1 | YME1-like 1 (S. cerevisiae) | 1.986 |
| NM_175893 | DKFZP564J0863 | DKFZP564J0863 protein | 1.98 |
| NM_002799 | PSMB7 | proteasome (prosome, macropain) subunit, beta type, 7 | 1.98 |
| BC030224 | CD48 | CD48 antigen (B-cell membrane protein) | 1.979 |
| NM_005619 | RTN2 | reticulon 2 | 1.979 |
| NM_022137 | SMOC1 | SPARC-related modular calcium binding 1 | 1.977 |
| XM_059140 | dJ39G22.2 | dJ39G22.2 (novel protein) | 1.976 |
| NM_005926 | MFAP1 | microfibrillar-associated protein 1 | 1.975 |
| NM_006711 | RNPS1 | RNA binding protein S1, serine-rich domain | 1.974 |
| NM_004582 | RABGGTB | Rab geranylgeranyltransferase, beta subunit | 1.971 |
| NM_176889 | TAS2R49 | taste receptor, type 2, member 49 | 1.959 |
| NM_024902 | FLJ13236 | hypothetical protein FLJ13236 | 1.958 |
| NM_004134 | HSPA9B | heat-shock 70kDa protein 9B (mortalin-2) | 1.952 |
| NM_138395 | METRS | methionine-tRNA synthetase | 1.949 |
| NM_019016 | KRT24 | keratin 24 | 1.943 |
| AK022028 | C1orf43 | chromosome 1 open reading frame 43 | 1.942 |
| NM_003139 | SRPR | signal recognition particle receptor ('docking protein') | 1.94 |
| AF348999 | MTM | metallothionein M | 1.939 |
| NM_012399 | PITPNB | phosphatidylinositol transfer protein, beta | 1.933 |
| NM_012286 | MORF4L2 | mortality factor 4-like 2 | 1.927 |
| XM_036589 | KIAA1078 | KIAA1078 protein | 1.926 |
| NM_013417 | IARS | isoleucine-tRNA synthetase | 1.923 |
| NM_018428 | HCA66 | hepatocellular carcinoma-associated antigen 66 | 1.922 |
| NM_032578 | MYPN | myopalladin | 1.917 |
| NM_139279 | MCFD2 | multiple coagulation factor deficiency 2 | 1.914 |
| NM_001536 | HRMT1L2 | HMT1 hnRNP methyltransferase-like 2 (S. cerevisiae) | 1.914 |
| NM_006430 | CCT4 | chaperonin containing TCP1, subunit 4 (delta) | 1.913 |
| NM_014050 | MRPL42 | mitochondrial ribosomal protein L42 | 1.908 |
| NM_006392 | NOL5A | nucleolar protein 5A (56kDa with KKE/D repeat) | 1.907 |
| NM_003288 | TPD52L2 | tumor protein D52-like 1 | 1.907 |
| NM_025151 | RCP | opsin 1 (cone pigments), long-wave-sensitive (color blindness, protan) | 1.904 |
| NM_080655 | MGC17337 | similar to RIKEN cDNA 5730528L13 gene | 1.901 |
| NM_020162 | DHX33 | DEAH (Asp-Glu-Ala-His) box polypeptide 33 | 1.898 |
| NM_006298 | ZNF192 | zinc finger protein 192 | 1.897 |
| NM_178167 | ZNF598 | zinc finger protein 598 | 1.892 |
| NM_018696 | ELAC1 | elaC homolog 1 (E. coli) | 1.89 |
| NM_001997 | FAU | Finkel-Biskis-Reilly murine sarcoma virus ubiquitously expressed (fox-derived); ribosomal protein S30 | 1.887 |
| XM_353083 | LOC388117 | hypothetical LOC388117 | 1.885 |
| NM_003143 | SSBP1 | single-stranded DNA binding protein 1 | 1.882 |
| NM_003710 | SPINT1 | serine protease inhibitor, Kunitz type 1 | 1.881 |
| NM_014171 | CRIPT | postsynaptic protein CRIPT | 1.874 |
| NM_173791 | PDZK8 | PDZ domain containing 8 | 1.874 |
| NM_004094 | EIF2S1 | eukaryotic translation initiation factor 2, subunit 1 alpha, 35kDa | 1.869 |
| NM_006660 | CLPX | ClpX caseinolytic protease X homolog (E. coli) | 1.859 |
| XM_290592 | HIP1R | huntingtin interacting protein-1-related | 1.858 |
| NM_015889 | PCQAP | PC2 (positive cofactor 2, multiprotein complex) glutamine/Q-rich-associated protein | 1.855 |
| NM_022917 | NOL6 | nucleolar protein family 6 (RNA-associated) | 1.852 |
| NM_015959 | TMX2 | thioredoxin-related transmembrane protein 2 | 1.847 |
| NM_001759 | CCND2 | cyclin D2 | 1.847 |
| NM_020240 | CDC42SE2 | CDC42 small effector 2 | 1.846 |
| NM_004849 | APG5L | APG5 autophagy 5-like (S. cerevisiae) | 1.844 |
| X58235 | ATP8B2 | ATPase, Class I, type 8B, member 2 | 1.843 |
| NM_005339 | HIP2 | huntingtin interacting protein 2 | 1.843 |
| NM_004078 | CSRP1 | cysteine and glycine-rich protein 1 | 1.843 |
| NM_145062 | C6orf113 | chromosome 6 open reading frame 113 | 1.842 |
| NM_015470 | RAB11FIP5 | RAB11-family interacting protein 5 (class I) | 1.84 |
| NM_024894 | FLJ14075 | hypothetical LOC79954 | 1.836 |
| NM_144711 | MGC2610 | hypothetical protein MGC2610 | 1.83 |
| BC009943 | GPSM1 | G-protein signaling modulator 1 (AGS3-like, C. elegans) | 1.825 |
| NM_018491 | CBWD1 | COBW domain containing 1 | 1.823 |
| NM_004398 | DDX10 | DEAD (Asp-Glu-Ala-Asp) box polypeptide 10 | 1.822 |
| NM_013323 | SNX11 | sorting nexin 11 | 1.821 |
| NM_002817 | PSMD13 | proteasome (prosome, macropain) 26S subunit, non-ATPase, 13 | 1.819 |
| BC012148 | DENR | density-regulated protein | 1.816 |
| NM_016451 | COPB | coatomer protein complex, subunit beta | 1.816 |
| AK023887 | ANKRD11 | ankyrin-repeat domain 11 | 1.813 |
| NM_000498 | CYP11B2 | cytochrome P450, family 11, subfamily B, polypeptide 2 | 1.811 |
| NM_005998 | CCT3 | chaperonin containing TCP1, subunit 3 (gamma) | 1.81 |
| NM_006710 | COPS8 | COP9 constitutive photomorphogenic homolog subunit 8 (Arabidopsis) | 1.8 |
| AF186109 | TPM4 | tropomyosin 4 | 1.799 |
| NM_019095 | C20orf155 | chromosome 20 open reading frame 155 | 1.799 |
| NM_006392 | NOL5A | nucleolar protein 5A (56kDa with KKE/D repeat) | 1.798 |
| NM_015702 | C2orf25 | chromosome 2 open reading frame 25 | 1.795 |
| NM_031422 | CHST9 | carbohydrate (N-acetylgalactosamine 4-0) sulfotransferase 9 | 1.793 |
| NM_014397 | NEK6 | NIMA (never in mitosis gene a)-related kinase 6 | 1.791 |
| NM_002092 | GRSF1 | G-rich RNA sequence binding factor 1 | 1.786 |
| NM_003784 | SERPINB7 | serine (or cysteine) proteinase inhibitor, clade B (ovalbumin), member 7 | 1.782 |
| NM_152913 | DKFZp761L1417 | hypothetical protein DKFZp761L1417 | 1.781 |
| NM_015436 | RCHY1 | ring finger and CHY zinc finger domain containing 1 | 1.772 |
| NM_002938 | RNF4 | ring finger protein 4 | 1.766 |
| NM_003017 | SFRS3 | splicing factor, arginine/serine-rich 3 | 1.766 |
| NM_002018 | FLII | flightless I homolog (Drosophila) | 1.763 |
| NM_080605 | B3GALT6 | UDP-Gal:betaGal beta 1,3-galactosyltransferase polypeptide 6 | 1.755 |
| AK054652 | ARL5 | ADP-ribosylation factor-like 5 | 1.754 |
| AL833897 | MYLK | myosin, light polypeptide kinase | 1.752 |
| NM_006330 | LYPLA1 | lysophospholipase I | 1.751 |
| NM_031298 | MGC2963 | hypothetical protein MGC2963 | 1.751 |
| NM_000588 | IL3 | interleukin 3 (colony-stimulating factor, multiple) | 1.75 |
| NM_003127 | SPTAN1 | spectrin, alpha, non-erythrocytic 1 (alpha-fodrin) | 1.75 |
| NM_006496 | GNAI3 | guanine nucleotide-binding protein (G protein), alpha-inhibiting activity polypeptide 3 | 1.744 |
| NM_182631 | LOC348840 | hypothetical protein LOC348840 | 1.74 |
| NM_021062 | HIST1H2BB | histone 1, H2bb | 1.738 |
| NM_000426 | LAMA2 | laminin, alpha 2 (merosin, congenital muscular dystrophy) | 1.734 |
| AL834353 | KIAA1043 | KIAA1043 protein | 1.731 |
| NM_005499 | UBA2 | SUMO-1 activating enzyme subunit 2 | 1.729 |
| NM_001675 | ATF4 | activating transcription factor 4 (tax-responsive enhancer element B67) | 1.729 |
| NM_022044 | SDF2L1 | stromal cell-derived factor 2-like 1 | 1.728 |
| NM_018362 | LIN7C | lin-7 homolog C (C. elegans) | 1.727 |
| NR_001541 | TTTY5 | testis-specific transcript, Y-linked 5 | 1.722 |
| NM_001892 | CSNK1A1 | casein kinase 1, alpha 1 | 1.715 |
| **NM_021141** | **XRCC5** | **X-ray repair complementing defective repair in Chinese hamster cells 5 (double-strand-break rejoining; Ku80)** | **1.715** |
| NM_012456 | TIMM10 | translocase of inner mitochondrial membrane 10 homolog (yeast) | 1.714 |
| NM_006114 | TOMM40 | translocase of outer mitochondrial membrane 40 homolog (yeast) | 1.711 |
| NM_003372 | VBP1 | von Hippel-Lindau binding protein 1 | 1.708 |
| NM_002925 | RGS10 | regulator of G-protein signaling 10 | 1.706 |
| NM_020859 | ShrmL | Shroom-related protein | 1.703 |
| NM_001658 | ARF1 | similar to dJ133P16.1 (ADP-ribosylation factor 1) | 1.702 |
| NM_014793 | LCMT2 | leucine carboxyl methyltransferase 2 | 1.702 |
| NM_006870 | DSTN | destrin (actin depolymerizing factor) | 1.699 |
| NM_017656 | ZNF562 | zinc finger protein 562 | 1.698 |
| NM_012325 | MAPRE1 | microtubule-associated protein, RP/EB family, member 1 | 1.697 |
| NM_015888 | HOOK1 | hook homolog 1 (Drosophila) | 1.696 |
| NM_005585 | SMAD6 | SMAD, mothers against DPP homolog 6 (Drosophila) | 1.696 |
| NM_032016 | STARD3NL | STARD3 N-terminal like | 1.694 |
| NM_004733 | SLC33A1 | solute carrier family 33 (acetyl-CoA transporter), member 1 | 1.694 |
| NM_020865 | DHX36 | DEAH (Asp-Glu-Ala-His) box polypeptide 36 | 1.691 |
| XM_036408 | CHR2SYT | family with sequence similarity 62 (C2 domain containing) member B | 1.688 |
| NM_004305 | BIN1 | bridging integrator 1 | 1.688 |
| BC032822 | EPB41L5 | erythrocyte membrane protein band 4.1 like 5 | 1.687 |
| NM_012110 | CHIC2 | cysteine-rich hydrophobic domain 2 | 1.684 |
| NM_000785 | CYP27B1 | cytochrome P450, family 27, subfamily B, polypeptide 1 | 1.684 |
| NM_182513 | Spc24 | spindle pole body component 24 homolog (S. cerevisiae) | 1.681 |
| NM_138446 | C7orf30 | chromosome 7 open reading frame 30 | 1.676 |
| NM_006379 | SEMA3C | sema domain, immunoglobulin domain (Ig), short basic domain, secreted, (semaphorin) 3C | 1.675 |
| NM_024111 | MGC4504 | hypothetical protein MGC4504 | 1.674 |
| NM_006275 | SFRS6 | splicing factor, arginine/serine-rich 6 | 1.672 |
| NM_020939 | CPNE5 | copine V | 1.672 |
| NM_016013 | NDUFAF1 | NADH dehydrogenase (ubiquinone) 1 alpha subcomplex, assembly factor 1 | 1.672 |
| NM_033086 | FGD3 | FYVE, RhoGEF and PH domain containing 3 | 1.671 |
| NM_006601 | TEBP | thyroid transcription factor 1 | 1.669 |
| NM_031314 | HNRPC | heterogeneous nuclear ribonucleoprotein C (C1/C2) | 1.669 |
| BC032771 | FLJ13105 | methylenetetrahydrofolate dehydrogenase (NADP+ dependent) 2-like | 1.666 |
| NM_002112 | HDC | headcase homolog (Drosophila) | 1.666 |
| NM_002669 | PLRG1 | pleiotropic regulator 1 (PRL1 homolog, Arabidopsis) | 1.664 |
| NM_001881 | CREM | cAMP responsive element modulator | 1.655 |
| NM_006253 | PRKAB1 | protein kinase, AMP-activated, beta 1 non-catalytic subunit | 1.655 |
| NM_001851 | COL9A1 | collagen, type IX, alpha 1 | 1.644 |
| NM_007204 | DDX20 | DEAD (Asp-Glu-Ala-Asp) box polypeptide 20 | 1.642 |
| NM_003633 | ENC1 | ectodermal-neural cortex (with BTB-like domain) | 1.641 |
| NM_033550 | TP53RK | TP53-regulating kinase | 1.64 |
| NM_006993 | NPM3 | nucleophosmin/nucleoplasmin, 3 | 1.638 |
| NM_015525 | IBTK | inhibitor of Bruton agammaglobulinemia tyrosine kinase | 1.638 |
| NM_001829 | CLCN3 | chloride channel 3 | 1.638 |
| NM_016507 | CRK7 | CDC2-related protein kinase 7 | 1.637 |
| NM_017825 | ADPRHL2 | ADP-ribosylhydrolase like 2 | 1.632 |
| NM_002376 | MARK3 | MAP/microtubule affinity-regulating kinase 3 | 1.63 |
| NM_003872 | NRP2 | NEL-like 2 (chicken) | 1.63 |
| NM_005548 | KARS | lysyl-tRNA synthetase | 1.628 |
| NM_005605 | PPP3CC | protein phosphatase 3 (formerly 2B), catalytic subunit, gamma isoform (calcineurin A gamma) | 1.627 |
| NM_021154 | PSAT1 | phosphoserine aminotransferase 1 | 1.626 |
| NM_017847 | C1orf27 | chromosome 1 open reading frame 27 | 1.626 |
| NM_178517 | PIGW | phosphatidylinositol glycan, class W | 1.626 |
| NM_015607 | DKFZP547E1010 | DKFZP547E1010 protein | 1.622 |
| NM_005628 | SLC1A5 | solute carrier family 1 (neutral amino acid transporter), member 5 | 1.619 |
| AB046773 | KIAA1553 | KIAA1553 | 1.619 |
| NM_014928 | HSHIN1 | HIV-1-induced protein HIN-1 | 1.614 |
| NM_018299 | FLJ11011 | hypothetical protein FLJ11011 | 1.613 |
| NM_000373 | UMPS | uridine monophosphate synthetase (orotate phosphoribosyl transferase and orotidine-5'-decarboxylase) | 1.613 |
| **NM_002879** | **RAD52** | **RAD52 homolog (S. cerevisiae)** | **1.611** |
| **NM_002745** | **MAPK1** | **mitogen-activated protein kinase 1** | **1.611** |
| NM_152711 | LOC399978 | hypothetical gene supported by BC031979 | 1.61 |
| NM_144652 | LETM2 | leucine zipper-EF-hand containing transmembrane protein 2 | 1.61 |
| NM_002266 | KPNA2 | karyopherin alpha 2 (RAG cohort 1, importin alpha 1) | 1 605 |
| NM_173510 | FLJ33814 | hypothetical protein FLJ33814 | 1 605 |
| NM_013326 | C18orf8 | chromosome 18 open reading frame 8 | 1 604 |
| NM_006481 | TCF2 | transcription factor 2, hepatic; LF-B3; variant hepatic nuclear factor | 1 603 |
| NM_003648 | DGKD | diacylglycerol kinase, delta 130kDa | 1 603 |
| AF143885 | PACSIN2 | protein kinase C and casein kinase substrate in neurons 2 | 1 602 |
| NM_139021 | ERK8 | mitogen-activated protein kinase 15 | 1 601 |
| NM_002787 | PSMA2 | proteasome (prosome, macropain) subunit, alpha type, 2 | 1.6 |
| NM_002134 | HMOX2 | heme oxygenase (decycling) 2 | 1 599 |
| NM_052943 | FAM46B | family with sequence similarity 46, member B | 1 598 |
| NM_007214 | SEC63 | SEC63-like (S. cerevisiae) | 1 597 |
| NM_032869 | CML66 | NudC domain containing 1 | 1 596 |
| NM_019644 | ANKRD7 | ankyrin repeat domain 7 | 1 593 |
| **NM_002874** | **RAD23B** | **RAD23 homolog B (S. cerevisiae)** | **1 591** |
| NM_018149 | FLJ10587 | hypothetical protein FLJ10587 | 1 589 |
| NM_015942 | CGI-12 | CGI-12 protein | 1 585 |
| NM_001177 | ARL1 | aldo-keto reductase family 1, member B10 (aldose reductase) | 1 582 |
| NM_145648 | SLC15A4 | solute carrier family 15, member 4 | 1.581 |
| NM_016626 | RKHD2 | ring finger and KH domain containing 2 | 1.58 |
| NM_006588 | SULT1C2 | sulfotransferase family, cytosolic, 1C, member 2 | 1.58 |
| **NM_007266** | **XAB1** | **XPA binding protein 1** | **1.578** |
| NM_024831 | NCOA6IP | nuclear receptor coactivator 6 interacting protein | 1.578 |
| NM_003081 | SNAP25 | synaptosomal-associated protein, 25kDa | 1.573 |
| NM_032331 | ECE2 | endothelin converting enzyme 2 | 1.573 |
| NM_005692 | ABCF2 | ATP-binding cassette, sub-family F (GCN20), member 2 | 1.57 |
| NM_013375 | ABT1 | activator of basal transcription 1 | 1.57 |
| NM_001569 | IRAK1 | interleukin-1 receptor-associated kinase 1 | 1.57 |
| NM_012238 | SIRT1 | sirtuin (silent mating type information regulation 2 homolog) 1 (S. cerevisiae) | 1.568 |
| NM_013352 | SART2 | squamous cell carcinoma antigen recognized by T cells 2 | 1.568 |
| NM_003819 | PABPC4 | poly(A)-binding protein, cytoplasmic 4 (inducible form) | 1.567 |
| NM_007033 | RER1 | RER1 retention in endoplasmic reticulum 1 homolog (S. cerevisiae) | 1.567 |
| NM_032810 | ATAD1 | ATPase family, AAA domain containing 1 | 1.566 |
| NM_015934 | NOP5/NOP58 | nucleolar protein NOP5/NOP58 | 1.565 |
| NM_002175 | IFNA21 | interferon, alpha 21 | 1.564 |
| NM_001625 | AK2 | adenylate kinase 2 | 1.563 |
| NM_172006 | WFDC10B | WAP four-disulfide core domain 10B | 1.563 |
| NM_005063 | SCD | stearoyl-CoA desaturase (delta-9-desaturase) | 1.558 |
| NM_052998 | ODC-p | ornithine decarboxylase-like | 1.557 |
| NM_030578 | MGC4093 | hypothetical protein MGC4093 | 1.556 |
| NM_000403 | GALE | UDP-galactose-4-epimerase | 1.555 |
| NM_032116 | KATNAL1 | katanin p60 subunit A-like 1 | 1.552 |
| NM_018471 | LEREPO4 | likely ortholog of mouse immediate early response, erythropoietin 4 | 1.551 |
| NM_017958 | PLEKHB2 | pleckstrin homology domain containing, family B (evectins) member 2 | 1.548 |
| NM_002842 | PTPRH | protein tyrosine phosphatase, receptor type, H | 1.547 |
| NM_053034 | ANTXR1 | anthrax toxin receptor 1 | 1.547 |
| NM_018838 | DAP13 | 13kDa differentiation-associated protein | 1.546 |
| NM_024021 | MS4A4A | membrane-spanning 4-domains, subfamily A, member 4 | 1.544 |
| NM_016045 | C20orf45 | GNAS complex locus | 1.539 |
| NM_004429 | EFNB1 | ephrin-B1 | 1.537 |
| NM_005772 | RCL1 | RNA terminal phosphate cyclase-like 1 | 1.533 |
| NM_022768 | RBM15 | RNA binding motif protein 15 | 1.533 |
| NM_020437 | LOC57168 | similar to aspartate beta hydroxylase (ASPH) | 1.532 |
| NM_014248 | RBX1 | ring-box 1 | 1.531 |
| NM_024520 | FLJ22555 | hypothetical protein FLJ22555 | 1.531 |
| NM_006023 | C10orf7 | chromosome 10 open reading frame 7 | 1.53 |
| NM_018093 | FLJ10439 | hypothetical protein FLJ10439 | 1.53 |
| NM_014550 | CARD10 | caspase recruitment domain family, member 10 | 1.53 |
| NM_002786 | PSMA1 | proteasome (prosome, macropain) subunit, alpha type, 1 | 1.53 |
| NM_174925 | LOC205251 | hypothetical protein LOC205251 | 1.527 |
| NM_032479 | MRPL36 | mitochondrial ribosomal protein L36 | 1.524 |
| NM_000607 | ORM1 | orosomucoid 1 | 1.521 |
| NM_015523 | DKFZP566E144 | small-fragment nuclease | 1.52 |
| NM_004225 | MFHAS1 | malignant fibrous histiocytoma amplified sequence 1 | 1.519 |
| NM_006590 | USP39 | ubiquitin-specific protease 39 | 1.519 |
| NM_005880 | DNAJA2 | DnaJ (Hsp40) homolog, subfamily A, member 2 | 1.515 |
| NM_015001 | SPEN | RNA-binding motif protein 15 | 1.514 |
| L03172 | IGKV1-5 | immunoglobulin kappa variable 1-5 | 1.511 |
| NM_019618 | IL1F9 | interleukin 1 family, member 9 | 1.505 |
| NM_014503 | DRIM | down-regulated in metastasis | 1.505 |
| NM_033225 | CSMD1 | CUB and Sushi multiple domains 1 | 1.503 |
| NM_018000 | FLJ10116 | likely ortholog of mouse dilute suppressor | 1.502 |
| NM_005057 | RBBP5 | retinoblastoma binding protein 5 | 1.502 |
| NM_145001 | STK32A | serine/threonine kinase 32A | 1.501 |
| NM_003387 | WASPIP | Wiskott-Aldrich syndrome protein interacting protein | 1.5 |
